# Designing of future ornamental crops: a biotechnological driven perspective

**DOI:** 10.1093/hr/uhad192

**Published:** 2023-09-25

**Authors:** Mahinder Partap, Vipasha Verma, Meenakshi Thakur, Bhavya Bhargava

**Affiliations:** Floriculture Laboratory, Agrotechnology Division, Council of Scientific and Industrial Research (CSIR), Institute of Himalayan Bioresource Technology (IHBT), Post Box No. 6, 176 061 (HP) Palampur, India; Academy of Scientific and Innovative Research (AcSIR), Ghaziabad, 201002, Uttar Pradesh, India; Floriculture Laboratory, Agrotechnology Division, Council of Scientific and Industrial Research (CSIR), Institute of Himalayan Bioresource Technology (IHBT), Post Box No. 6, 176 061 (HP) Palampur, India; Floriculture Laboratory, Agrotechnology Division, Council of Scientific and Industrial Research (CSIR), Institute of Himalayan Bioresource Technology (IHBT), Post Box No. 6, 176 061 (HP) Palampur, India; Floriculture Laboratory, Agrotechnology Division, Council of Scientific and Industrial Research (CSIR), Institute of Himalayan Bioresource Technology (IHBT), Post Box No. 6, 176 061 (HP) Palampur, India; Academy of Scientific and Innovative Research (AcSIR), Ghaziabad, 201002, Uttar Pradesh, India

## Abstract

With a basis in human appreciation of beauty and aesthetic values, the new era of ornamental crops is based on implementing innovative technologies and transforming symbols into tangible assets. Recent advances in plant biotechnology have attracted considerable scientific and industrial interest, particularly in terms of modifying desired plant traits and developing future ornamental crops. By utilizing omics approaches, genomic data, genetic engineering, and gene editing tools, scientists have successively explored the underlying molecular mechanism and potential gene(s) behind trait regulation such as floral induction, plant architecture, stress resistance, plasticity, adaptation, and phytoremediation in ornamental crop species. These signs of progress lay a theoretical and practical foundation for designing and enhancing the efficiency of ornamental plants for a wide range of applications. In this review, we briefly summarized the existing literature and advances in biotechnological approaches for the improvement of vital traits in ornamental plants. The future ornamental plants, such as light-emitting plants, biotic/abiotic stress detectors, and pollution abatement, and the introduction of new ornamental varieties via domestication of wild species are also discussed.

## 1. Introduction

Human senses and aesthetic feelings have been attracted to ornamental plants for millennia, producing numerous new cultivars [1]. Despite challenges posed by SARS-CoV-2 and the global pandemic, the annual flower trade reached 5.6 billion euros in 2021 [2]. In terms of annual revenue, the European Union is the leading producer and consumer of both potted and cut flower ornamentals (carnations, chrysanthemums, orchids, gerbera, freesia, lilies, gladiolus, roses, and tulips) [3, 4]. The main objective of ornamental plant cultivation is to produce new attractive cultivars with enhanced flowering and other aesthetic qualities/floral traits for commercial usage [1]. Several plant breeding techniques have been adopted to enhance or improve color, plant architecture, shelf life, and abiotic/biotic stress resistance. Although cross-breeding and mutation breeding have resulted in the development of numerous cultivars, they are only pertinent to a small number of floral characteristics. In addition, conventional breeding, hybridization, and mutation approaches have several drawbacks, including high heterozygosity and subtractive one-point improvement.

Given that this century is widely recognized as the dawn of the biotechnology-driven bioeconomy. The conventional plant breeding methods merged with transgenic technology can help to create desired phenotypes in flowers, stress resilience, and postharvest longevity in ornamental plants that are not seen in nature [5]. Genetic transformation is a potent biotechnological tool that may be utilized to produce an “additive” one-point improvement, as opposed to mutation breeding, which produces a “subtractive” one-point improvement. Since the first report of genetic transformation in ornamental plants in 1987, and till date, a large number of ornamental plants have been transformed [6, 7]. Despite its potential utility, only a few kinds of genetically modified ornamental crops have been field tested, and the only genetically engineered ornamental crops now available in the market are color-modified carnation and rose variants [6]. Blue-colored carnations, roses, and chrysanthemums are examples of genetically modified ornamental crops that could not have been bred possibly by conventional techniques [7]. Among ornamental plants, genetic modification was maximally executed in chrysanthemum (26.7%), followed by petunia (15.2%), orchids (6.75%), rose (6.7%), dianthus (5.5%), etc. [8]. In addition, since the introduction of next-generation sequencing technology, genomic sequence data of potential ornamental plant species have been reported in just a few years. The availability of genome sequence data facilitates not only the comprehension of genome structure and function, but also the study of the genesis and evolution, mapping, and cloning of the genes responsible for essential floral features [5, 8–11]. In parallel to the classic genetic modification techniques, genome editing technology, such as the clustered regularly inter-spaced short palindromic repeats (CRISPR), has developed for targeted (knockout/in, base and prime editing) genetic modification in the genome of plants. As the major approach for determining gene function and developing novel cultivars, it is predicted that cutting-edge genome editing technologies like CRISPR-associated 9 (CRISPR/Cas9) will expedite crop breeding [12–16]. The CRISPR/Cas9 technique was employed to alter the genome of *Petunia hybrida*, making it the inaugural instance of an ornamental plant genome being modified. This modification aimed to facilitate the production of novel pigments. So far, different floral attributes (senescence, flowering, pigment, and self-incompatibility) have been modified among ornamental crops (chrysanthemum, dendrobium, torenia, ipomoea, lilium, and phalaenopsis) through CRISPR/Cas technology, but the major emphasis is on the development of novel color varieties [13]. These research findings suggested that CRISPR/Cas9-induced gene editing (GE) is effective in ornamental plants for a novel trait introduction and its enhancement.

Recently, light-emitting, biotic/abiotic stress detectors, and pollution-abating plants are among the prospective ornamentals that can be developed using genetic engineering and nanotechnology [17–19]. In addition, Meher et al. [20] and Cao et al. [21] offer unique techniques for producing gene-edited plants without the use of plant tissue culture. These biotechnological advances have the potential for *de novo* domestication of wild ornamental plant species and the ability to develop novel ornamental traits with a vast array of applications [1, 22]. During domestication, target DNA sequences are inserted into the crop of interest via bioballistics, *Agrobacterium,* and other novel techniques (CRISPR, RNAi). The process of *de novo* domestication through genetic modification can be divided into four distinct phases. Firstly, potential genes that may have played a role in the domestication of crops are identified. These genes should have orthologs that can be edited in a closely related wild species. Secondly, targeted genetic modifications are carried out on these specific loci in the wild species. Thirdly, genotypic and phenotypic traits that are desirable are screened. Finally, agronomical evaluations are conducted to assess the overall performance and suitability of the genetically modified species [22].

In this regard, there are a number of reviews out there on this topic, but none of them have compiled a thorough overview of both existing and newly emerging biotechnological approaches, such as GE and *de novo* domestication, in the context of enhancing floral attributes for developing and designing new varieties/cultivars of ornamental crops with enormous market potential. This paper also discusses the development of future-generation ornamental traits in plants such as phytoremediation, light emission, and stress biosensing, in addition to upgrading classic ornamental traits (sensory or aesthetic). Future research potential in this sector is highlighted, along with the pitfalls and challenges associated with deploying biotechnological methods to create ornamental plants with the traits of interest.

Therefore, the review article delivers an up-to-date and comprehensive discussion about the molecular, biotechnological, and omics perspectives for designing future ornamental crops and enhancing their commercial important traits. The present review study information holds significant value in informing the development of viable strategies for research communities, industries, and commercial sectors involved in the cultivation of future ornamental plants. These strategies aim to cater to diverse purposes throughout the Anthropocene epoch.

## 2. Genome sequencing

**Figure 1:**
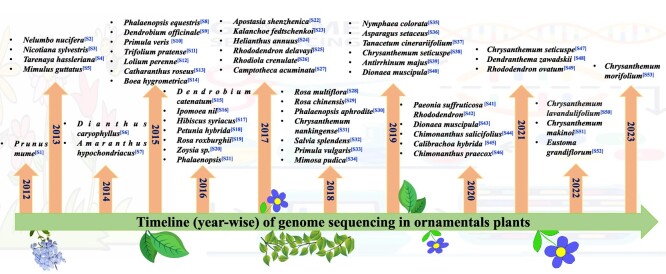
Chronological (timeline) depiction of ornamental plant species whose genomes have been sequenced. The data in superscript represent the relevant citation for a related plant species. The citations are provided in the supplementary file S1 (see references S1–S53).

The genome sequence data are helpful for conducting research on gene evolution, genomic variants, gene regulation, and other significant biological systems based on information on the full genome sequence [11]. The timeline of genome sequencing in major ornamental plant species is represented in Fig. 1 (for references, see supplementary file S1; S1–S53). *Prunus mume* was the first ornamental plant to have its entire genome sequenced, and this was accomplished in 2012 [5, 23]. On the basis of the genome sequence of prunus, 7813 differentially expressed genes (DEGs) were identified, which provided a unique perspective on the formation of floral scent. After that, the genomes of more than 65 different ornamental plant species have been sequenced in the past decade [24, 25]. The field of ornamental horticulture has experienced significant advancements in molecular biology research, primarily attributed to the outcomes of whole-genome sequencing conducted on several plant species within this domain [26]. The investigation of genome structure and function in ornamental horticulture not only contributes to comprehension but also holds significant implications for the exploration of ornamental plant evolution, functional gene mapping, and cloning for important traits, thus speeding up ornamental plant breeding [27].

China is recognized as the “mother of gardens” due to its abundance of materials pertaining to attractive ornamental plants. Furthermore, China has successfully accomplished or taken the lead in conducting genome sequencing for a total of 32 distinct species of ornamental plants [5]. Nevertheless, it is worth noting that both Japan and the USA have successfully conducted genome sequencing on over 10 distinct ornamental species. The size of the complete genome of ornamental plants that have been sequenced can range from 237 megabytes (MB) to 13.79 gigabytes (GB), and the scaffold N50 can be anywhere from 13.8 kilobytes (KB) to 65.35 MB [5, 28]. The genome-based phylogenetic trees that have been created by Zheng et al. [5] for all of the species were made public and are classified into 21 orders and 35 families. When it comes to ornamental plants, the groups Rosaceae, Orchidaceae, and Asteraceae are considered to be the most representative in terms of high-quality sequencing. Roses are the most widely cultivated decorative and aromatic plants in the world; therefore, their cultural and economic significance cannot be overstated. The complete genome sequence of *Rosa multiflora* was determined using the utilization of Illumina MiSeq and HiSeq technologies [29]. This accomplishment was achieved by Japanese researchers who were interested in deciphering the molecular mechanism behind the flower color, scent, and growth characteristics. The size of the *Rosa* genome is 560 MB, and its N50 contig is 24 MB, thus making it one of the most thorough plant genomes. Similarly, *Rosa chinensis*, which was a doubled haploid line derived from “Old-Blush,” has been sequenced and republished by Hibrand Saint-Oyant et al. [30]. In the Orchidaceae family, the whole-genome sequence of *Phalaenopsis equestris* has been completed with a scaffold N50 size of 359.1 KB*.* Species of *P. equestris* were the first monocot flower to have its whole genome sequenced [31]. This *Phalaenopsis* sp. holds substantial importance as an ornamental potted plant and possesses considerable economic value on a global scale. Researchers from China and Australia collaborated to assemble a draft genome that was 3.1 GB in size and belonged to an important winter-flowering *Phalaenopsis* cultivar known as KHM190. A different species of the genus *Phalaenopsis (Phalaenopsis aphrodite)* was also subjected to high-quality genome sequencing in 2018 [32]. The scaffold N50 size for this species was 19.7 MB. Both the *Dendrobium officinale* and *Dendrobium catenatum* entire genomes were subjected to additional scrutiny by researchers from China [33, 34]. The genome size of *Apostasia shenzhenica* was estimated to be roughly 800 MB, while its scaffold N50 size was determined to be 288 KB. Additionally, the observed heterozygosity of this species was found to be 1.14% [35]. With a total length of 549 MB, scaffold N50 of 41 MB, and 41 264 projected genes, the chromosome-level genome assembly of *Rhododendron ovatum* (azalea) has been reported by Wang et al. [36]. This study presents a high-quality chromosome-level reference genome of the evergreen azalea, offering novel insights into the mechanisms underlying low-altitude adaptability and flower scent enabled by tandem duplications [36]. The *de novo* whole-genome assembly of *Chrysanthemum nankingense* and *Chrysanthemum seticuspe* was sequenced in 2018 and 2019, respectively [37, 38]. In another Asteraceae species (*Chrysanthemum morifolium*), the genome size was determined to be greater than 9 GB [39]. The genome assembly and annotation of *C. morifolium*, which consists of 27 pseudochromosomes (8.15 GB; scaffold N50 of 243.69 MB), has revealed the origin and evolution of cultivated chrysanthemums [40]. In sunflower, 97% of annotated genes have been linked to a total of 17 pseudo chromosomes, making the genome 2.94 GB in size [5]. The resequencing of various species based on genome-wide association studies was made easier to aid the identification of critical genomic areas related to plant domestication and selection. The genome-wide resequencing thus enables the researchers to analyze genetic resources, uncover genetic variations, analyze genetic evolution, and ultimately help in the prediction of relevant candidate genes behind important floral traits.

## 3. Candidate genes associated with important ornamental traits

**Figure 2:**
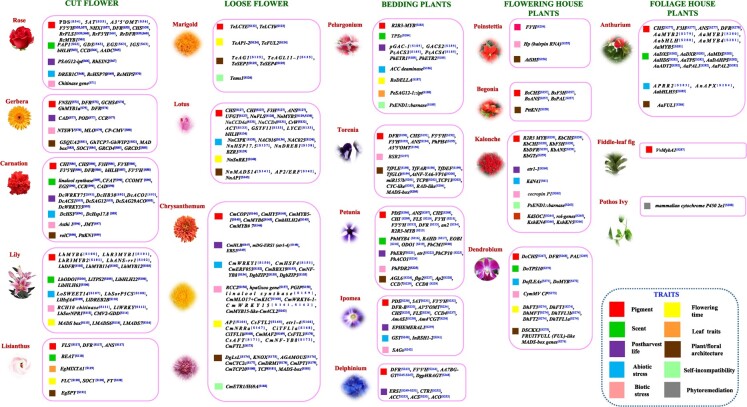
List of candidate genes involved in the regulation or improvement of important traits in different ornamental plant species (cut flower, loose flower, bedding plants, flowering house plants, and foliage house plants). The data in superscript represent the relevant citation for the associated gene. The citations are provided in the supplementary file S1 (see references S54–S288).

The important ornamental traits such as flower morphology, anatomy, flower pigmentation, floral scent biosynthesis, dormancy release, abiotic and biotic stress tolerance, early flowering, and postharvest quality are discussed below along with their candidate genes associated with these traits (Fig. 2) (see supplementary file S1; references S54–S288).

### 3.1. Plant architecture

The morphological appearance and architecture of ornamental plants are also one of the determining factors for their commercial acceptance by consumers. The dwarf phenotype in chrysanthemum was achieved through the heterologous expression of the mutant gai gene, which is known to be insensitive to gibberellic acid in *Arabidopsis*. This resulted in a diminished sensitivity to gibberellin and subsequently led to the production of a dwarf chrysanthemum [41]. Plant architecture was altered when the phytochrome *B1* gene from tobacco (*Nicotiana tabacum*) was ectopically expressed in chrysanthemum. The transgenic plants were shorter and had wider branch angles than their wild-type counterparts. To create a dwarf chrysanthemum, researchers used an RNAi expression vector to silence the genes responsible for brassinosteroids and gibberellins biosynthesis [42]. By overexpressing homeotic genes *KxhKN4* and *KxhKN541* isolated from *Kalanchoe × houghtonii*, scientists were able to produce ornamental *Kalanchoe blossfeldiana* plants with a more compact stature [43]. By expressing *ROL* genes from *Agrobacterium rhizogenes*, transgenic gypsophila, carnation, and rose were created with enhanced rooting [44]. In addition, overexpression of the gene for cytokinin biosynthesis (*ipt*; isopentenyl transferase) in chrysanthemum increased the plant’s tendency to produce more branches [45]. Five gerbera transgenic plants were successfully generated by *Agrobacterium*-mediated transformation using *GSQUA2* (a gerbera *SQUAMOSA* like gene) under the control of the *35 S* CaMV promoter [46]. Dwarf chrysanthemum can be easily developed through the coordinated silencing of the *DmCPD* and *DmGA20ox* genes [47]. A novel strategy for the generation of chrysanthemum cultivars with a reduced number of tillers was proposed using *DgD27*, a cloned gene from *Dendranthema grandiflorum* [48].

### 3.2. Flower morphology and anatomy

There are two categories of genes involved in flower growth and development. The genes that direct the development of inflorescence meristems and the subsequent growth of flowers constitute one group. Mutations in these genes can cause early or late flowering in plants by affecting their ability to develop inflorescence meristems or flower meristems, respectively. Homeoboxes arise from mutations in the second category of genes, which control floral organ development. *CAG* gene transfer in the antisense orientation into *C. morifolium* was also shown [49]. It was discovered that gynoecium and androecium are transformed into corolla-like tissues when the *CAG* gene is suppressed. However, the rate of change was minimal, and it was limited to a modification of floral morphology [44]. Transgenic gerbera plants with an overabundance of *GSQUA2* showed similar pronounced modifications in their vegetative structures, such as an elongation of their vegetative axis. These plants were discovered to be sensitive with poor root development and sparse flowering [50]. In lily’s *LMADS1-M* gene caused more flowers to form on leafy stems than in the wild [51, 52]. Moreover, the floral structure of transgenic *Lisianthus* flowers has been altered. In this, the second whorl of petals on transgenic plants morphed into structures resembling sepals, and the third whorl’s stamens were distorted. It is worth noting that transgenic carnations containing the *rol C* gene under the control of the *35S CaMV* promoter produced more flowers and a greater yield after pinching [53]. The generation of a multipetal phenotype in *Torenia fournieri* through the dual knockdown or elimination of the *TfPLENA (TfPLE)* and *TfFALINELLI (TfFAR)* genes using RNA interference (RNAi) or CRISPR/Cas9-mediated genome editing has been reported [54]. The number of petals in flowers notwithstanding, the size of petals plays a significant role in plant reproduction and horticulture. This characteristic is principally determined by the process of cell growth. The inhibition of petal extension in *Gerbera hybrida* is mediated by the transcription factor *GhTCP7*. This transcription factor interacts with the WIP-type zinc finger protein GhWIP2, leading to the activation of *GhIAA26*. The *GhIAA26* is responsible for encoding an auxin signaling regulator [55].

In *Chrysanthemum lavandulifolium*, six *CmCYC2* genes were shown to have expression restricted to petal ray florets [56]. Overexpressing *CmCYC2c* in chrysanthemum, on the other hand, increased both the length of the ray florets and the total number of flowers produced. This suggests that flower shape is highly complicated and is inherited by polygenic mechanisms, as no discernible alteration was observed. Two *Cyc2CL* genes, *Cyc2CL-1* and *Cyc2CL-2*, were discovered by Liu et al. [57] to have crucial functions in the formation of chrysanthemum stamens and ray florets. Chrysanthemum anemone-type flower shapes appear to be determined by a combination of additive and epistatic quantitative trait loci, which have been identified. According to a recent study conducted by Shen et al. [58], it is hypothesized that the molecular control underlying the evolutionary transition from a radiating to a disciform capitulum in chrysanthemum may have been facilitated by the malfunctioning of *CYC2g* orthologs. When it comes to flower formation, the TCP family of transcription factors (TFs) plays a significant role. Class I TCP TF member *CmTCP20* has been identified, and it has been linked to petal elongation [59]. Through transcriptome analysis, Liu et al. [60] found around 1800 DEGs, some of which were regulatory genes involved in floral meristem and organ development in chrysanthemum florets. Chrysanthemum “Anastasia Dark Green” bud sport analysis of petal abnormalities uncovered a network of genes that controlled the shape of hooked petals. The overexpression of the *CmYAB1* gene, which is a homolog of the chrysanthemum polarity gene, led to the development of transgenic plants with decreased petal curvature and flat petals. These plants had inflorescence characterized by a pompon-like morphology [61]. One of the most well-known chrysanthemum inflorescence shapes is the anemone type, which displays colors and tubular flowers.

It is shown by the *MADS-box* genes, which regulate the timing of flowering and the maturation of several floral organs [62]. Transgenic chrysanthemums flowered prematurely due to the overexpression of *AP1-like* genes, which are part of the *MADS-box* gene family and are found in the Asteraceae [63]. In *G. hybrida*, TCPs (CYCLOIDEA [CYC]/TEOSINTE BRANCHED1-like) and *MADS-Box* transcription factors have demonstrated their role in regulating heteromorphic flower type identity [64]. Flowering and the transition from inflorescence meristems to floral meristems occurred earlier in transgenic dendrobium orchids. It has also been shown that *DOAP1*, an *AP1* ortholog, was overexpressed in transgenic dendrobium than wild types [65]. Similarly, transgenic orchids were able to earlier flower by overexpressing *MADS-box* genes such as *DOSOC1* and *OMADS1* [65]. Diploid wild chrysanthemum (*C. seticuspe*) was mined for three *FLOWERING LOCUS T (FT)*-like paralogues, and *CsFTL3* emerged as the dominant regulator of photoperiodic flowering [66]. The epidermal cells of ray floral petals seemed shorter, and the shape has been transformed when *Gh-SOC1* was overexpressed, but this did not affect the timing of flowering in *G. hybrida*. A lack of phenotypic alterations was noticed in transgenic gerbera plants after the downregulation of *Gh-SOC1* [50]. The gene *CmTCP20*, belonging to the teosinte branched 1/cycloidea/proliferating cell factors (TCPs) gene family, was subjected to overexpression in chrysanthemum plants. This genetic modification led to the development of larger flower inflorescences and elongated petals [59]. As a result of overexpression of *CmCYC2c*, the number of flowers produced by *C. lavandulifolium* and the length of the ligules on the petals of ray florets both increased dramatically. The genetically engineered *Epimedium grandiflorum* ectopically expressed the *MADS-box* gene (*LMADS1-M*) from the lily, which displayed altered floral transition and development (*Lilium longiflorum*) [52]. *Arabidopsis* chimeric repressors *AGSRDX48* and *TCP349* have been used in other investigations to alter floral characteristics in transgenic torenia [67]. In conclusion, the scientific community and the general public can gain a deeper understanding of the efficacy of genetic engineering of ornamental plants through molecular breeding approaches, which permit critical examination of biological processes for floral modifications.

### 3.3. Flower pigment and color

Structural and regulatory genes associated with various pigments such as flavonoids, anthocyanins, carotenoids, and betalains have been characterized and expressed to attain novel flower color in ornamental plants. The study revealed that there was a heightened expression of the genes responsible for dihydroflavonol 4-reductase and 3-*O*-glucosyltransferase in *H5* cultivars, which exhibit pink flowers. However, no such rise in gene expression was observed in Keikai or Jinba cultivars, which display white flowers [42]. The expression of *chalcone synthase*, *chalcone isomerase, flavanone 3-hydroxylase*, and *flavonoid 3′-hydroxylase* was seen in all floral samples. There has been a proposition positing that the absence of anthocyanin in white flowering cultivars cannot be attributed to any obstruction in the manifestation of their genetic expression [42]. Delphinidin, one of several types of anthocyanidins, is made up of 40% of the total in the blue-tinged petals of transgenic flowers. The delphinidin-based anthocyanins are responsible for the production of violet/blue variants in many flowering plant species, including roses, carnations, and chrysanthemums. However, due to the absence of flavonoid 3′,5′-hydroxylase (*F3′5′H*), delphinidin-based anthocyanins biosynthesis genes was downregulated [68]. Research on flower color variation has proven to be more fruitful when using suppression technology like RNAi, cosuppression, or antisensemediated silencing [7]. The *F3′5′H* gene was used to induce delphinidin accumulation in roses and other ornamental cultivars, changing their color to violet or purple [69, 70]. Overexpression of *PhF3′5′H* caused a color shift from pink to pale purple in the lilium [71, 72]. Putting the pansy *F3′5′H* gene under the control of a piece of the rose chalcone synthase promoter led to a considerable enhancement of delphinidin production in the transgenic chrysanthemum petals [60]. Synchronized expression of *PhF3′5′H* and *HyDfr* produced a deep purple hue, in contrast to individual expression. This suggests a possible function for the *HyDfr* gene in promoting the synthesis of delphinidin [72]. Intriguingly, the insertion of the butterfly pea *uridine diphosphate (UDP) glucose: anthocyanin-3′,5′-O-glucosyltransferase* gene into chrysanthemum resulted in the development of blue blossoms [73]. There is a gene, *CmCCD4a*, which is highly specific to the white chrysanthemum ray petal. This is a single dominant gene that prevents the accumulation of carotenoids in flower petals [74]. From this, we can deduce that white flowers’ lack of *CmCCD4a* causes the breakdown of synthetic carotenoids into a colorless chemical. With the help of homology analysis and functional classification, researchers have found 84 candidate genes involved in pigment biosynthesis. The transcriptome data also revealed the presence of genes encoding transcription factors involved in the regulation of specialized metabolism, including *myb,* erythroid-derived factor, *WD40* family member, *WRKY*, nuclear anchoring complex, basic helix–loop–helix, and basic zipping factor [75]. Improved pigment accumulation and induction of cyanidin synthesis were two outcomes of *GMYB10* overexpression in transgenic gerbera plants [76]. *PAP1*-transgenic roses, which have had the *Arabidopsis* (*PAP1*) transcription factor introduced in *Rosa hybrida*, have been shown to generate anthocyanin at a higher level and to accumulate more eugenol compound [77]. In chrysanthemum, *CmMYB#7*, an R3 MYB transcription factor *CmMYB#7*, regulates anthocyanin biosynthesis by competing with *CmMYB6*, which, along with *CmbHLH2*, is involved in anthocyanin synthesis activation [78, 79]. Scientists also discovered that mitotically heritable epigenetic changes (methylation and demethylation) of *CmMYB6* govern anthocyanin production in chrysanthemums [78]. The overexpression of *RcMYB1*, a major transcription factor belonging to the *R2R3-MYB* family, has been observed to result in a notable increase in anthocyanin accumulation in white rose petals [80].

### 3.4. Flower scent biosynthesis

The gene known as *benzylalcohol acetyltransferase (BEAT)* has the potential to directly facilitate the production of benzyl acetate, a prominent compound responsible for the flowery scent in *P. mume* [81]. Furthermore, a total of 44 unique *PmBEATs* were discovered in *P. mume* through the analysis of genomic data obtained from both *P. mume* and *Prunus persica*. It was observed that the overexpression of either the *PmBEAT36* or *PmBEAT37* genes resulted in an increase in benzyl acetate production inside the petal protoplasts of *P. mume*, thereby leading to the generation of the distinctive floral fragrance [81]. Research has shown that the floral scent of *Lilium* “Siberia” could be efficiently regulated by *LibHLH22* and *LibHLH63* [82]. To make the petals of lisianthus flowers fragrant, the *Clarkia breweri* gene *BEAT* was introduced [83]. In petunia, *CHORISMATE MUTASE* (*PhCM1*) gene has a crucial role in the biosynthesis of benzenoids/phenylpropanoids (FVBP), which are responsible for floral fragrance. These FVBP compounds have been found to be reduced by approximately 60%–70% in petunias expressing *PhCM1*RNAi [84]. Two terpene synthase genes responsible for floral scent synthesis in Lilium “Siberia” (*LoTPS2* and *LoTPS4*) have been cloned and functionally characterized in *Arabidopsis* [85]. The variety of other genes has been identified, which regulates the floral scent biosynthesis in various commercially important ornamental crops such as lily (*LhODO1*), rose (*AADC*, *COMT, MYB1*), carnation (*linalool synthase*), pelargonium (*TPS*), petunia (*BAHD*, *EOB1*), and dendrobium (*DoTPS10*) (Fig. 2).

#### 3.4.1. Controlling dormancy

Dormancy break and cold-induced flowering in *P. mume* were investigated at the molecular level by Zhao et al. [86]. Six dormancy-associated *MADS box* (*DAM*) genes (*PmDAM1* to *PmDAM6*) were discovered to have tandem repeats throughout the genome. In bulbous crops such as lilium, the expression of certain genes, known as DEGs, was observed to be influenced by low-temperature settings. These DEGs are associated with antioxidant activity, epigenetic modification, and transcription factors. The aforementioned genes comprised an intricate regulatory mechanism for the activation of dormancy [86]. The expression levels of DAMs are regulated by the binding of C-repeat binding factors (CBFs), which are genes associated with cold response. This binding occurs specifically to the DRE/CRT (dehydration-responsive element/C-repeat) *cis*-acting region present in the promoters of DAMs. This study proposes a molecular regulatory model elucidating the role of the *PmDAM* and *PmCBF* genes in the regulation of flower bud dormancy and its release in response to low-temperature signals. These studies reveal that six *PmCBFs* and *PmDAM4-694* work together to regulate cold-induced dormancy, demonstrating a sense-response link between these two types of genes [87]. It is well known that flowering and vegetative growth in geophytes depend on bud dormancy. Several investigations have confirmed the role of TF’s in regulating corm dormancy [88, 89]. Cold storage represses ABA production and signaling in *Gladiolus hybridus* promoting corm dormancy release (CDR). In *Gladiolus*, *GhNAC83* was identified as a transcription factor that promotes ABA synthesis and inhibits CK biosynthesis pathways, and its silencing promoted CDR [90]. Additionally, in *Gladiolus*, a transcription factor *GhBPC2* (*BASIC PENTACYSTEINE2*) was identified that epigenetically regulated CDR [91].

#### 3.4.2. Biotic stress resistance

Many ornamental plants now have a resistance to bacterial and fungal infections, which can be attributed to the introduction of genes encoding for chitinase, glucanase, osmotin, defensin, and other proteins. Transgenic ornamental plants have been evaluated and shown to be more resistant to biotic stressors than their wild counterparts [92]. The introduction of a rice *chitinase* gene increased the plant’s resistance to powdery mildew in roses [93]. In *D. grandiflorum* cv. Shinba, three *N*-methyl transferase genes (*CaXMT1*, *CaMXMT1*, and *CaDXMT1)* were introduced, which resulted in a significant decrease in *Botrytis cinerea* infection [94]. Transgenic chrysanthemum with enhanced resistance to Septoria leaf spot disease was also supported by *chiII* [94]. Agrobacterium-mediated transformation has been utilized to develop transgenic lilium lines, resulting in increased resistance to *B. cinerea*. This resistance is attributed to the ectopic expression of a rice chitinase gene [95]. In comparison to their natural ancestors, transgenic lilium plants that express *OcID86* have a 75% reduction in the parasitic impacts of *Pratylenchus* [96].

Increased resistance to aphids was observed in transgenic chrysanthemums that overexpressed either the transcription factor *CmWRKY4817* or the protease inhibitor [97]. Increased resistance to *Fusarium oxysporum* was also seen in transgenic gladiolus plants that expressed the synthetic antimicrobial peptide *D4E1* [98]. Transgenic *Ornithogalum* plant tissues expressing the antimicrobial peptide *tachyplesin I* showed a significant reduction in bacterial proliferation, colonization, and disease signs. Incorporating the antimicrobial protein gene *ace-AMP1* into *R. hybrida* has increased the plant’s resistance to powdery mildew [99]. T4 lysozyme, a class II chitinase, a1, 3 glucanase, a type I ribosome-inhibiting protein (RIP), and another antifungal gene were all used to create transgenic roses with antifungal properties [44, 93]. Transplanting the rice *chitinase* gene in roses has recently improved the plant’s resistance to powdery mildew [93]. Transposing the polygalacturonase-inhibiting protein (*PGIP*) gene from *P. mume* into chrysanthemum increased its resistance to the *Alternaria* leaf spot [100]. Many transgenic chrysanthemum cultivars were created by Shinoyama et al. [101] by inserting a mutated *cry1A*b gene. The expression of the gene has shown an insecticidal effect against tobacco budworms and common cutworms (*Spodoptera litura*). In petunia, *PhPDR2* is the main factor that controls the amount of petuniasterone in the leaves and trichomes, which helps the plant survive herbivory [102]. The development of resistance to *Puccinia horiana* Henn. in chrysanthemum is facilitated by the *WRKY15-1* gene through the activation of the salicylic acid signaling system [103, 104]. The previous study has also documented and demonstrated the modulation of the immune system by the *CmWRKY6–1–CmWRKY15-like* cascade response to *F. oxysporum* infection in chrysanthemum [105]. Another study conducted in chrysanthemum showed that in response to aphid (*Macrosiphoniella sanborni*) feeding, *CmMYB15-like-Cm4CL2* regulates lignin formation and cell wall thickening in chrysanthemum, thus increasing insect resistance in a *Cm4CL2*-dependent way [106].

### 3.5. Abiotic stress resistance

Scientists study genetically modified ornamental plants in an effort to boost their resilience toward abiotic stress. When subjected to abiotic challenges, ornamental plants also react in a variety of ways [107]. Rose species, for instance, are known to be especially vulnerable to cold stress. The *MtDREB1C* gene from Medicago was used to confer tolerance to freezing stress in *R. chinensis*. The susceptibility to freezing stress in petunia plants was found to be increased as a result of the silencing of *PhDREB1F* or *PhDREB1I* through virus-induced gene silencing. In addition, the downregulation of *PhDREB1F* or *PhDREB1I* resulted in a reduction in the expression levels of *PhZFP1* and *PhGolS1-1* [108]. Drought stress can be reduced by adjusting water loss and epidermal morphology. *CmNF-YB8*, a nuclear factor Y (NF-Y) B-type component, affects chrysanthemum drought resistance through changing stomatal state and leaf cuticle thickness. *CmNF-YB8-RNAi* transgenic chrysanthemum lines were more drought tolerant than control lines, but *CmNF-YB8-OX* lines were less drought tolerant [109]. For the purpose of improving the chrysanthemum tolerance to salinity and water shortage, two constructs, *35S: DREB1A* and *rd29A: DREB1A*, were inserted into the genome. In comparison to the *35S: DREB1A* transgenic plants, the *rd29A: DREB1A* plants exhibited greater tolerance to both salt and drought [110]. The constitutive gene expression of *CcSOS1*, which encodes plasma Na+/H+ antiporter, improved the salt stress tolerance of chrysanthemum [111]. Transgenic chrysanthemum plants overexpressing *CmWRKY17* (a transcription repressor) were more sensitive to salt than their wild-type counterparts [97]. The *bZIP* transcription factor has also shown its role in regulating genes responsible for resistance against abiotic stresses like drought, salt, and low-temperature stress. Researchers have demonstrated the role of *DgbZIP3* for the cold stress tolerance in chrysanthemum (*C. morifolium* Ramat.) [112]. The red-flowered variety of *R. chinensis* (Jacq.) is highly preferred. However, in low temperatures, these roses lose many of their shoots due to cold stress. This flower is thus not economically or ecologically viable in cold climates. China rose transgenics with the *Medicago truncatula* (*Mt*) *DREB1C* gene showed improved freezing tolerance with no observable morphological or developmental abnormalities [107].

### 3.6. Flowering time

Floral development is impossible without the *MADS-box* genes, which regulate both flowering time and the growth of floral organs [51, 52]. Exogenous *LFY* overexpression encourages early flowering, as shown by *Agrobacterium*-mediated transformation in *Sinningia* spp. [113]. In transgenic gloxinia plants, altering *miRNA159* expression by either overexpressing or suppressing it caused flowers to bloom either later or earlier than expected. The up- or downregulation of *SsGAMYB* depends upon the degree of *miR159a* expression during flowering. Therefore, it was determined that *miR159*-mediated *GAMYB* expression also plays a significant function in regulating the flowering time of ornamentals [114]. The *CsFTL3* gene (Flowering Locus T-like paralog) in *C. seticuspe* has been discovered to function as a photoperiodic flowering regulator [115]. An antiflorigen gene from a wild *C. seticuspe* is largely induced in leaves under noninductive conditions. Gain- and loss-of-function investigations showed that *CsAFT* systematically inhibits flowering and dominates the obligatory photoperiodic response [116]. The suppression of *CmNF-YB8*, a nuclear factor gene, in the short day plant *C. morifolium*, leads to an accelerated shift from the juvenile to adult phase, as well as premature flowering, irrespective of the duration of daylight [117]. The transition from vegetative to reproductive growth is most likely the most critical developmental switch for a flowering plant. In summer-flowering chrysanthemum, the *BBX8-FT* regulatory module is an essential factor of reproductive development [118]. Research using cold treatment in lily bulbs showed numerous potential candidate genes involved in vernalization process [119, 120]. The *GSQUA2* gene hastened the flowering process in gerbera [46]. Overexpression of *GhSOC1* caused a little reduction in flower individuality. The overexpression altered the petal shape and size of the epidermis but had no effect on the ray flower’s blossoming time. *Gh-SOC1* downregulation, on the other hand, did not result in any appreciable phenotypic change in transgenic plants [50]. When compared to nontransformed *Lisianthus* spp., those that had *OMADS1* inserted into their genome bloomed much sooner and produced more flowers. *FT* (*Blooming locus T*) is the primary integrator of several flowering genes that respond to environmental cues like light, temperature, and more [121]. Overexpression of the *MADS-box* (*OMADS1*) gene resulted in early flowering in transgenic orchids (*Oncidium*) [122]. Increasing the expression of flowering genes such *AP1*, *Lfy*, and *SOC1*, *FT* promotes early flowering among ornamental (Fig. 2).

#### 3.6.1. Postharvest quality

Several biotechnological approaches have been employed to extend flower longevity. Promoting a rise in cytokinin levels is an important way to delay plant senescence. Transforming petunia flowers with the enzyme *PSAG12-IPT*, which catalyzes the first step in the biosynthesis of cytokinins, resulted in an overproduction of cytokinins, which delayed flower senescence and reduced the flowers’ sensitivity to the hormone ethylene [123]. In a study conducted by Zakizadeh et al. [124], the researchers investigated the effects of transforming the potted rose cultivar “Linda” using *Agrobacterium tumefaciens* containing a *PSAG12-ipt* construct. The results revealed that transgenic plants, when subjected to darkness and treated with exogenous ethylene, exhibited a substantial 8-fold elevation in the expression levels of the *ipt* gene. The targeted activation of the *ipt* gene has been observed to effectively postpone the process of leaf senescence and improve the plant’s ability to withstand the effects of externally applied ethylene. Many ornamental plants have genes encoding essential enzymes in the ethylene pathway inserted into them to prevent the ageing process from occurring. Resistance to ethylene can be maintained by inhibiting the genes responsible for ethylene biosynthesis, resulting in a longer shelf life. The ethylene sensitivity in modified chrysanthemums was diminished through the production of a mutant ethylene receptor gene (*mDG-ERS1s*) [125]. Carnation plants engineered to express an antisense *ACC oxidase* gene produced less ethylene, and their petals remained fresh for a longer time [1126]. Furthermore, by turning off the *ACO* gene (involved in ethylene synthesis), transgenic carnation plants with delayed petal senescence have been reported [126]. *ETR1* ethylene receptors and *EIN3* ethylene transcription factors are examples of ethylene pathway components that are promising in this regard. In 1999, researchers developed transgenic carnations that contained the *etr1-1* gene from *Arabidopsis* [127]. Transgenic carnation flowers had a 3-fold increase in vase life compared to the control. Modifying the ethylene receptor gene has been found effective in extending the vase life of *Oncidium* and *Odontoglossum* [128]. Lignin biosynthesis is a crucial process for stem mechanical strength and postharvest quality of cut flower. The manipulation of genes involved in the lignin synthesis pathway, namely, peroxidase (*POD*), cinnamyl alcohol dehydrogenase (*CAD*), and cinnamoyl-CoA reductase (*CCR*), resulted in a decrease in stem bending disorder observed in cut gerbera (*Gerbera jamesonii*) flowers [129]. Lignin production in chrysanthemums is regulated by the unusual *bHLH* protein *CmHLB*. When compared to the wild type, *CmHLB* overexpression in chrysanthemum dramatically increased mechanical stem strength, cell wall thickness, and lignin content [130].

## 4. CRISPR/Cas technology

The CRISPR/Cas9 system, also known as the clustered regularly interspaced short palindromic repeats/CRISPR-associated protein 9, has gained significant popularity in the field of crop development. This can be attributed to its user-friendly nature, streamlined design, and enhanced efficacy in targeting both single and multiple genes [131]*.* Among ornamentals, *Lotus japonicus* was the primary plant to undergo genome editing with CRISPR/Cas9 technology. Single and multiple gene mutant plants were generated by targeting genes involved in symbiotic nitrogen fixation, including symbiosis receptor-like kinase and leghaemoglobin loci (*LjLb1–3*) [132]. The nodule-specific *LjLb2* promoter has been demonstrated to be just as effective as the constitutively active *CaMV35S* promoter for GE in nodules. Genome editing was performed using a transgenic chrysanthemum that expresses a mutant version of the yellow-green fluorescent protein (*CpYGFP*) gene [133]. Chrysanthemum genome editing was proposed using the *PcUbi* as Cas9 promoter and *AtU6* as *sgRNA* promoter. In a recent study, a UV–visible reporter-assisted CRISPR/Cas9 GE system has been developed for the alteration of flowering time in *Chrysanthemum indicum* [134]. In order to get rid of tanshinones from plants while reducing the impact on other phenolic compounds, the diterpene synthase (*SmCPS1*) gene responsible for tanshinone production was mutated using CRISPR/Cas9 technology in *Salvia miltiorrhiza* [135]. This genetic modification resulted in the emergence of chimeric mutants exhibiting complete albinism, light-yellow pigmentation, and albino-green pigmentation, following the editing of the PDS locus in two lilium species. *Petunias* also exhibited an albino phenotype after being transformed with a CRISPR/Cas9 construct that eliminates the *PDS* gene [16]. Mutant lines of petunia *PhACO1* that were created by CRISPR/Cas9 editing bloomed for far longer than their wild-type counterparts. The *RhEIN2* gene, which encodes a key ethylene signaling pathway component, was modified using improved CRISPR/Cas9 technology. This genetic alteration made roses ethylene insensitive [136]. Editing the *Ipomoea nil* carotenoid cleavage dioxygenase (*CCD*) gene with CRISPR/Cas9 increased petal carotenoid content [16]. Mutations in the flavanone 3-hydroxylase (*F3′H*) gene caused frequent pale blue blooms in gene-edited torenia plants [137]. CRISPR/Cas9-mediated genome editing in *T. fournieri* was employed to study how a *RADIALIS-like gene* (*TfRAD1*) affects shape of petals, corolla uniformity, and pigmentation pattern. Unnaturally shaped and colored flowers were produced by gene-edited plants of torenia, which had loss-of-function alleles (*TfRAD1)* [138]. Recent research has shown that *T. fournieri MADS-box* mutants have an increased petal number. The observed phenomenon in the double biallelic plants, wherein stamens and carpels converted into petal-like structures, implies an integrated and synergistic function of the two genes in the development of reproductive organs. This is in contrast to the sole knockout of the *PLENA* gene (*TfPLE*), which solely induced morphological alterations in the carpels [54]. By employing two different multiplexing techniques, researchers were able to knock out three MADS genes in the orchid *P. equestris* that play important roles in flower initiation and development [139]. Recently, in *I. nil*, the *EPHEMERAL1 (EPH1)* gene has been successfully knocked out using the CRISPR/Cas9 system. Flowers on T-DNA-free biallelic T1 mutant plants wilted later than those on wild-type plants [140]. The *ACO1* gene, which codes for the ethylene-producing enzyme 1-aminocyclopropane-1-carboxylate oxidase, has been modified using GE to produce mutant *P. hybrida* plants with longer vase life [141]. CRISPR/Cas9-mediated genome editing reduced lignocellulose in *D. officinale* by targeting *C3H, C4H, 4-Coumarate: Coenzyme A Ligase, CCR, and Irregular Xylem5 (IRX)* genes [142]. Using CRISPR/Cas system, the *PiSSK1* gene, which encodes the Skp1 subunit of the SCFSLF complex, was knocked out in *Petunia inflata*, a plant known for its self-incompatibility [143]. Poinsettias that have undergone targeted mutation of the flavonoid 3′-hydroxylase gene using CRISPR/Cas9 have experienced a change in color [144]. The CRISPR/Cas9 technology has the potential to alter several traits in other ornamental varieties for a wide range of applications.

## 5. Plant tissue culture and gene delivery advances

Numerous studies on direct and indirect organogenesis, protoplast culture, somatic embryogenesis, protocorm development, micropropagation, and the effect of culture conditions on ornamental plant species have been conducted successfully so far [145–147]. The protoplast culture was carried out successfully on orchids, lilies, roses, chrysanthemums, petunias, carnations, coneflowers, and geraniums [148]. Somatic embryogenesis has been documented in numerous ornamental plant species such as roses, chrysanthemums, lilies, jasmine, lisianthus, carnations, camellias, cineraria, coneflowers, clematis, cypress, cyclamen, bellflower, passion flower, daisy, tulip, periwinkle, peony, and anthurium [147–149]. The embryo rescue approach was utilized to achieve interspecific hybridization and development of haploids as well as double haploids for stress tolerance in chrysanthemums, rose, tulip, lisianthus, lily, primula, cactus, gentian, begonia, carnation, gypsophila, and cyclamen [150]. Through the process of somatic hybridization, various beneficial traits such as high yield, disease, and abiotic stress resistance have been enabled. Various ornamentals, including roses, dendrobium, chrysanthemum, dianthus, iris, lily, petunia, calibrachoa, hydrangea, cyclamen, coneflowers, and saintpaulia, have been improved genetically by somatic hybridization [151]. According to a recent study, *Cymbidium aloifolium* exhibited the quickest seed germination, protocorm growth, and plantlet production in half Murashige and Skoog (MS) medium containing 6-benzylaminopurine [145]. For efficient bulb formation in tulips, methyl jasmonate (MJ) has been utilized in combination with other polyamines [152]. Chlorocholine chloride facilitated *in vitro* regeneration in phalaenopsis orchids. The inclusion of chlorine dioxide in the tissue culture media of chrysanthemum and gerbera resulted in an observed enhancement *in vitro* shoot and root regeneration, as reported in a study [147]. For orchids, gerbera, chrysanthemums, anthuriums, heliconias, lilies, giant protea, and hosta, the red LED was more effective for the callus induction/proliferation, shoot, and root organogenesis. However, far-red LED was effective for chrysanthemum plant development. The synergistic effect of red and blue light spectrum resulted in formation of protocorm-like bodies (PLBs) in phalaenopsis, and *in vitro* generation in rose, chrysanthemum, gerbera, anthurium, heliconia, peony, cymbidium, doritaenopsis, phalaenopsis, and calanthe was compatible in the combined effect of red LED and blue LED [147]. Blue LED stimulates shoot production in *D. officinale* and *Dendrobium kingianum* cultures. The efficacy of green LEDs in promoting *in vitro* regeneration was investigated in dendrobium and cymbidium species. In the case of *Dendrobium okinawense*, the exposure to green LED light resulted in production of PLBs, particularly when the culture media contained *p*-chlorophenoxyisobutyric acid. Both *D. okinawense* and *Bletilla ochracea* cultures have also been reported to benefit from yellow and orange LEDs for protocorm, shoot, plantlet regeneration, seed germination, and rhizoid development [147]. The review discussion on tissue culture in ornamental plants before the year of 2006 has been highlighted by Rout et al. [153].

**Figure 3:**
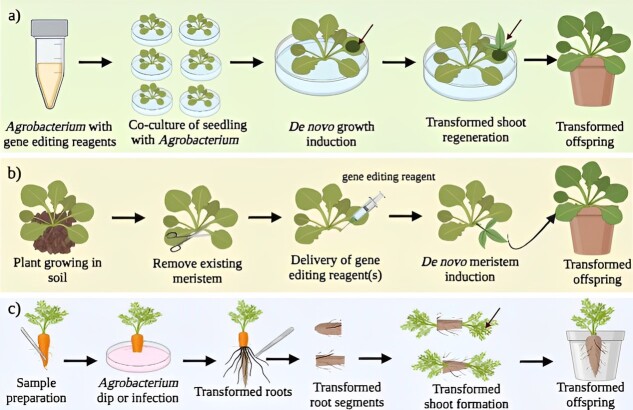
Stepwise representation of different methods used for the transformation of plant species. (a) *De novo* meristem induction under *in vitro* conditions, (b) *de novo* meristem induction under *in vivo* conditions, (c) CDB method. The image has been created in biorendor software.

The transformation or particle bombardment is a frequent method for delivering the desired gene into tissue-cultured plantlets or callus/cells. However, tissue culture is a time-consuming and expensive method that is required for genetic alteration even for individuals that are amenable to it. Recently, researchers have employed an easy approach referred to as the cut-dip budding (CDB) method to successfully transform many plant species, including herbaceous, tuberous, and woody plant species, without relying on plant tissue culture approach [21]. Researchers at the University of Minnesota have developed a new technique to create gene-edited plants without using tissue culture [154]. In this approach, *de novo* meristem induction was stimulated to generate shoots that carry the edited genes into future generations. The *de novo* induction of gene-edited meristems promises to circumvent a bottleneck in plant GE by eliminating the necessity for tissue culture. These possible approaches hold great potential to speed up the genetic engineering/GE process for developing new ornamental crops. The different methods used for the transformation of plant species with desired traits are represented in Fig. 3.

## 6. *De novo* domestication of wild ornamentals

**Figure 4:**
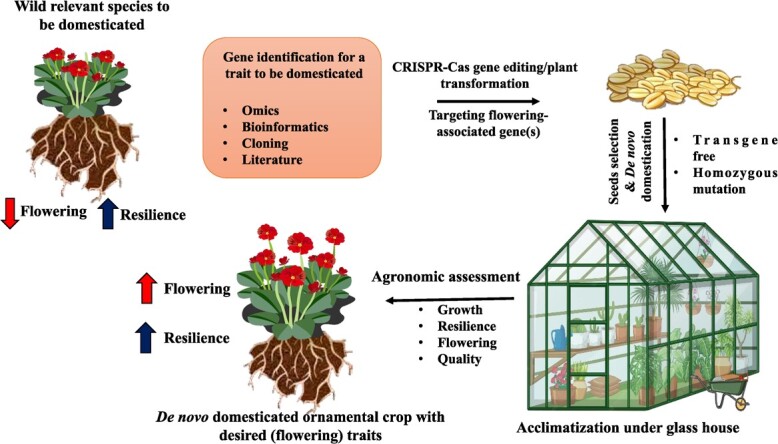
Domestication of ornamental wild species using GE approach. The figure depicts the *de novo* domestication of wild ornamental plant species by targeting genes associated with flowering traits.

The domestication of wild ornamental species is crucial for their commercial exploitation [155]. The floriculture industry can be benefited from the careful selection, cultivation, multiplication, and commercial exploitation of some important wild species with promising decorative value and resilience potential toward stresses. Angelonia, eragrostis, gaura, diascia, meconopsis, scaveola, sutera, scoparia, etc. are a few important domesticated floriculture species. Genetic engineering and genetic manipulations through induced mutation breeding accelerate the domestication process. It can be utilized for quick improvement of target-oriented wild/semiwild species for commercial exploitation to fulfill the demand and necessity of new ornamentals. The capacity to investigate the role of selection in domestication and any crop development has been greatly enhanced by next-generation sequencing. Reduced allelic diversity and dramatic shifts in gene expression and observable phenotype are hallmarks of domestication of the crop species we rely on. Future ornamental crops tailored to different climates and soil types can be developed through the domestication of wild species, allowing farmers to cultivate more land for longer periods of time. The recent progress in genetic engineering and the utilization of CRISPR/Cas technology has facilitated the swift process of domesticating wild plants *de novo* [156, 157]. The process of *de novo* domestication possesses the capacity to use beneficial traits exhibited by wild plants, thus circumventing the laborious procedures of crossbreeding and selective breeding necessary for conventional domestication, which relies on naturally occurring genetic modifications. *De novo* domestication of the orphan Solanaceae crop “groundcherry” (*Physalis pruinosa*) [158] and wild tomato [159] has recently been demonstrated. However, we know very little about the important genetic/epigenetic processes in most wild ornamental plants (particularly polyploidy species), which may differ significantly from those in well-studied model plants. Therefore, there are still many obstacles to overcome before the long-term goal of *de novo* domestication of wild ornamentals can be achieved, such as determining the genetic/epigenetic basis of desirable agronomic features in crops and wild plants and incorporating functional genomic discoveries with genome editing designs (Fig. 4).

## 7. New biotechnological innovations for designing future ornamental plants

### 7.1. Ornamental plants with phytoremediation potential

Indoor potted ornamental plants possess the ability to effectively eliminate airborne contaminants, thus presenting a promising ecological remedy for enhancing the quality of indoor air. Indoor potted ornamental plants are capable of removing pollutants from the air and serve as a potential green solution for the improvement of indoor air quality. Several species of predominantly ornamental plants have been studied and screened for their removal capability and efficiency toward air pollutants [160]. The biotechnological tools including genetic engineering and molecular biology have immense potential to transform or modify plants and microbes in concern of phytoremediation. Presently, the researchers aim to work on the genetic modification of common ornamental and houseplants for the improvement of indoor air quality. In this context, for elimination of chloroform and benzene from the indoor air, pothos ivy or devil’s ivy has been modified by genetic engineering approach by the expression of the *Mammalian Cytochrome P450 2e1* gene [161]. In the present era, engineered houseplants have the capability to degrade or accumulate the air pollutants. Inspired by nature, scientists focused on a protein called cytochrome *P450 2e1*, or *2e1*, which is present in all mammals, including humans. The main function of this enzyme *2e1*, which is found in the liver of human, is the conversion of benzene into phenol and chloroform into carbon dioxide and chloride ions. Therefore, instead of human body, researchers decided this reaction happens in plants, which is an example of the “green liver”. The expression of the *2e1* protein in plants can provide several advantages. Notably, this protein enables plants to utilize carbon dioxide and chloride ions for the synthesis of their food. Additionally, the presence of the 2e1 protein facilitates the utilization of phenol in the formation of the crucial components of plant cell walls. In addition, plants can be used as sensors to effectively monitor the environment, as they tend to absorb and sense various biotic/abiotic fluctuations in their surroundings. Plants that can sense/detect toxic chemicals, explosives, and environmental stresses have been successfully developed. Strano and Giraldo employed nanoparticles to improve plants’ photosynthesis and make them act as sensors against nitric oxide (a by-product of combustion) pollution.

### 7.2. Light-emitting plants for ornamental purpose

The development of light-emitting plants has been successfully accomplished through the use of advanced technologies such as genetic engineering and nanotechnology, all of which is covered in the following sections.

### 7.3. Nanobionic plants

**Figure 5:**
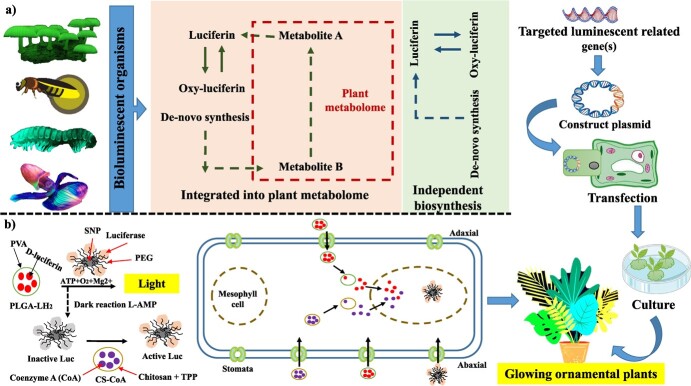
Schematic representation and different methods involved in the production of light-emitting plants. (a) Bioluminescence, integration of luciferin and oxyluciferin pathway into plant metabolome. (b) Nanobionic reaction mechanism of light production by firefly luciferase using nanoparticles.

Imagine that when it becomes dark, rather than turning on a lamp to read, you could use the light from a glowing plant that is placed on your desk. Previous attempts to generate plants that emit light focused on the genetic engineering of plants with the luciferase genes. However, this is a time-consuming process that only produces a very faint light as a result. These investigations were carried out on tobacco plants and *Arabidopsis thaliana*, both of which are model plants in the field of plant genetic research. The approach that was created in Strano’s lab, on the other hand, was applicable to any kind of plant, including watercress, arugula, kale, and spinach so far. Utilizing a strategy based on plant nanobionics, Kwak et al. [17] have made a significant advancement toward turning that vision into a functional reality. This new area of research aims to imbue plants with novel properties by encasing them in a variety of nanoparticles that interact with one another chemically. These nanoparticles include d-luciferin releasing poly(lactic-co-glycolic acid) (PLGA-LH_2_), coenzyme A functionalized chitosan (CS-CoA), semiconductor nanocrystal phosphors, and firefly luciferase conjugated silica (SNP-Luc). By using a technique known as pressurized bath infusion of nanoparticles, a combination of nanoparticles can be delivered to the stomata of a whole living watercress (*Nasturtium officinale*) system. Particles that were designed to collect in the extracellular space of the mesophyll and release luciferin and coenzyme A were developed, while the smaller particles containing the luciferase enter the cells that make up the mesophyll of the plant. The luciferin is gradually released from the PLGA particles, and it is then taken up by the plant cells, where the chemical reaction that causes the luciferin to glow is carried out by the luciferase enzyme. The initial efforts made by the researchers resulted in plants that could emit light for approximately 45 min. Since then, the researchers have enhanced this to 3.5 h. The researchers have also shown that they are able to extinguish the light by including nanoparticles that carry a luciferase inhibitor. Because of this, they might one day be able to develop plants that can turn off their light output in response to environmental factors such as exposure to sunshine. This technique might also be utilized to offer low-intensity indoor illumination or to transform ornamental plants into self-powered next-generation natural lights [17, 162]. The researchers anticipate that future iterations of this technique will allow them to paint or spray the nanoparticles onto plant leaves. This would make it possible to convert trees and other ornamental plants into sources of natural street lights (Fig. 5).

### 7.4. Autoluminescent plants

*Nicotiana tabacum* was the first plant with an autoluminescent system, and few transgenic plants had bacterial luciferase–luciferin pairs [163]. Due to limited light output, autoluminescent plants designed to express a bacterial bioluminescence gene cluster in plastids are not commonly used. In recent times, a novel eukaryote luciferase–luciferin system derived from fungi has been successfully incorporated into plant cells with the purpose of generating light [18, 164]. *Nicotiana tabacum* and *Nicotiana benthamiana* plants were created without external substrate by inserting fungal bioluminescence genes into their nuclear genomes. There is observable self-sustained luminosity in tobacco plants with a fungus-based bioluminescence system that converts caffeic acid into luciferin. Researchers have created genetically engineered autonomously illuminating *N. tabacum* plant by random-site genome integration of DNA cassettes containing codon-optimized versions of four *Neonothopanus nambi* bioluminescence gene(s) i.e., *nnluz* (luciferase), *nnhisps* (hispidin-synthase), *nnh3h* (hispidin-3-hydroxylase), and *nncph* (caffeoyl-pyruvate-hydrolase). This suggested that, unlike the bacterial bioluminescence system, the caffeic acid cycle does not harm plants or hinder growth. The naked eye could see light emission at all phases of development, with the intensity of the emission reaching its maximum in the blooms. They infused glowing plant leaves with luciferin or its predecessors to find light-limiting metabolites. After luciferin or hispidin injections, leaves exhibited strong light immediately, while caffeic acid supplementation produced lower intensity (Fig. 5). The fungi-based bioluminescent system is one of the potential optical molecular tools for plants due to its low cytotoxicity and high luminescent intensity [165]. 

### 7.5. Plants with fluorescence proteins or nanolanterns

To make ornamental luminous plants popular, fluorescence or bioluminescence should reach an adequate level of brightness. It is possible to develop fluorescent plant species by integrating the gene expression cassette of fluorescence proteins (FPs) into the genome. For instance, *CpYGFP*, a fluorescent protein derived from the sea plankton *Chiridius poppei*, can be activated at a laser line of 509 nm, and the emission can be captured at a wavelength of 517 nm, which is a red-shifted version of visible light [166]. Using an emission filter, it was possible to see green fluorescence emanating from the blooms of ornamental plants that had been genetically modified to include the *CpYGFP* gene. Additionally, *CpYGFP* derivatives (*eYGFP* and *eYGFPuv*) have been expressed in the flowers of a *P. hybrida*. The petunia flowers that were illuminated with visible and ultraviolet LEDs, respectively, generated green fluorescence [167]. A great number of FPs sustain their fluorescence stability in acidic circumstances. They stay very active in the intracellular environment of higher plants, which is acidic and has a pH range of about 4.5 to 7.2. Nanolanterns were implanted into plant cells in order to achieve an increase in fluorescence. Gene for green-enhanced nanolanterns was recently introduced into *A. thaliana*, and it was discovered that these lanterns may become brighter when excited by blue light. When the appropriate substrate furimazine was introduced, a remarkable enhancement of the luminescence occurred [168]. It has been observed that desiccated fluorescent flowers that were transplanted with the *CpYGFP* gene kept shining for more than a year after being embedded in fine-grained silica gels. This is due to the fact that some FPs are able to create fluorescence without the presence of water [169]. Glow-in-the-dark plants with luciferase–luciferin bioluminescent systems could be able to fulfill the requirements of this application.

## 8. Concluding remarks and future perspective

The present review reflects a wide range of studies performed on traits improvement in ornamental plants using both existing and advanced cutting-edge biotechnological approaches. A wider range of colors, flowering time, scent, longer vase life, male sterility, self-incompatibility, and resistance to abiotic/biotic stress are crucial traits to be improved for designing high-value ornamental plant species. By utilizing omics approaches, genomic data, genetic engineering, and GE tools, scientists have successively explored the underlying molecular mechanism and potential gene(s) behind floral induction, trait regulation, plant architecture, stress resistance, plasticity, adaptation, and phytoremediation in ornamental crop species. In this context, we have summarized up-to-date literature on genes associated with important traits in ornamental plant species. Using existing data, we reasonably say that introducing genetic modifications into ornamental plants is a logical and profitable idea from a scientific and economic standpoint. Moreover, the introduction of robust GE technology like CRISPR/Cas has considerable promise to facilitate the floriculture business through targeted genetic changes. Although there are many ways in which genetic engineering and genome editing might improve ornamental crop traits, there are still certain challenges that need to be overcome. Highly varied and genotype-dependent responses, with numerous recalcitrant cultivars, and diminished research efforts restrict the efficacy and usability of *in vitro* culture methods in a variety of ornamental species. The effectiveness of *in vitro* methods can be improved by optimizing various culture parameters, but this is a time-consuming process with outcomes that are often genotype dependent. Transformation methods that do not rely on plant tissue culture, such as in planta transformation, the CDB approach, *de novo* meristem induction, and the use of nanoparticle-mediated gene transfer, may circumvent these constraints. But there are other major pitfalls in utilizing the huge potential of GE technology for trait improvement among ornamentals, such as lack of genome or transcriptome data, high ploidy level, the complexity of the genome, and off-targeting. In various important ornamental crops, genome sequence is not available. The genomic resources are major prerequisite for the designing complementary gRNA sequences to drive Cas nucleases to the target location. Compared to other crops with simpler genomes, ornamental plants frequently have less access to high-quality genomic data since most ornamentals occur naturally as polyploids, and polyploid genomes are more challenging to sequence and annotate. Similar to polyploidy, heterozygosity is common in the genomes of ornamental plants. This complicates the use of genetic engineering and GE on such plants. Alleles from sequenced plants may be introgressed into unsequenced or unannotated ornamental plants to solve the heterozygosity issue in breeding. Another major concern is the negative public perception for transgenic crops, which are generally subjected to various domestic and international regulations. The gene editing have few advantages over transgenic approaches technology as it modifies endogenous genes within a plant and no foreign gene transfer is involved. Nevertheless, economic and legal constraints have slowed the spread of commercialization of genetically modified and gene edited crops in various nations, despite the promising scientific future of transgenic and gene-edited crops. To minimize this inconvenience, less stringent regulatory requirements for ornamental plants and other non-food plants are needed. Establishing a realistic, product-based, worldwide regulatory strategy is essential for accelerating the use of genetically modified and genome-edited technologies for designing future ornamental crops for commercial gains and boosting the economy.

## Supplementary Material

Web_Material_uhad192

## Data Availability

There are no new data associated with this article.
