# Supplementary material for: Designing of future ornamental crops: a biotechnological driven perspective
Source: Hortic Res. 2023 Sep 25;10(11):uhad192. doi: 10.1093/hr/uhad192 (PMC10681008; doi:10.1093/hr/uhad192)
Supplement: Web_Material_uhad192 [file web_material_uhad192.zip › Highlights.docx]

**Highlights:**

- Designing new varieties with desired traits is a primary aim of the ornamental industry
- Genetic engineering and CRISPR-cas technologies have the potential for trait improvement
- Domestication of wild ornamentals is a pre-requisite to obtaining novel varieties
- Light emitting and pollution-abating ornamentals are an evolving venture in ornamental plants
