# Supplementary material for: Designing of future ornamental crops: a biotechnological driven perspective
Source: Hortic Res. 2023 Sep 25;10(11):uhad192. doi: 10.1093/hr/uhad192 (PMC10681008; doi:10.1093/hr/uhad192)
Supplement: Web_Material_uhad192 [file web_material_uhad192.zip › supplimentary file S1.docx]

**Supplementary File S1**

**Title: Designing of future ornamental crops: A biotechnological driven perspective**

**Running title:** Biotechnological advancements in ornamental crops

Mahinder Partap^1,2,#^, Vipasha Verma^1,#^, Meenakshi Thakur^1,#^, Bhavya Bhargava^1,2^*

**^1^**Floriculture Laboratory, Agrotechnology Division, Council of Scientific and Industrial Research (CSIR)- Institute of Himalayan Bioresource Technology (IHBT), Post Box. No. 6, Palampur- 176 061 (HP), India

**^2^**Academy of Scientific and Innovative Research (AcSIR), Ghaziabad- 201002, Uttar Pradesh, India

***Corresponding Author:**

Dr. Bhavya Bhargava

Senior Scientist, Council of Scientific and Industrial Research (CSIR)- Institute of Himalayan Bioresource Technology (IHBT), Palampur- 176061 (HP), India

Email: [bhavya@ihbt.res.in;](mailto:bhavya@ihbt.res.in;) [bhavyaihbt@gmail.com](mailto:bhavyaihbt@gmail.com)

Telephone no.: +91-1894-233339; +91-9418373241; Fax: +91-1894-224433

**References of Fig. 1 (S1 to S53) and Fig. 2 (S54 to S288)**

**Fig. 1:** Chronological (timeline) depiction of ornamental plant species whose genomes have been sequenced. The superscript represent the relevant citation for a related plant species. The citations are provided in the supplementary file **(see references S1-S53)**.

1. Zhang Q, Chen W, Sun L, Zhao F, Huang B, Yang W, Tao Y, Wang J, Yuan Z, Fan G, Xing Z. The genome of *Prunus mume*. *Nat. Commun*. 2012 Jan;3(1):1318.
2. Ming R, VanBuren R, Liu Y, Yang M, Han Y, Li LT, Zhang Q, Kim MJ, Schatz MC, Campbell M, Li J. Genome of the long-living sacred lotus (*Nelumbo nucifera* Gaertn.). *Genome Biol.* 2013 May;14(5):1-1.
3. Sierro N, Battey JN, Ouadi S, Bovet L, Goepfert S, Bakaher N, Peitsch MC, Ivanov NV. Reference genomes and transcriptomes of *Nicotiana sylvestris* and Nicotiana tomentosiformis. *Genome Biol.* 2013 Jun;14(6):1-7.
4. Cheng S, van den Bergh E, Zeng P, Zhong X, Xu J, Liu X, Hofberger J, de Bruijn S, Bhide AS, Kuelahoglu C, Bian C. The *Tarenaya hassleriana* genome provides insight into reproductive trait and genome evolution of crucifers. *Plant Cell*. 2013 Aug 1;25(8):2813-30.
5. Hellsten U, Wright KM, Jenkins J, Shu S, Yuan Y, Wessler SR, Schmutz J, Willis JH, Rokhsar DS. Fine-scale variation in meiotic recombination in *Mimulus inferred* from population shotgun sequencing. *Proc. Natl. Acad. Sci. U.S.A.* 2013 Nov 26;110(48):19478-82.
6. Yagi M, Kosugi S, Hirakawa H, Ohmiya A, Tanase K, Harada T, Kishimoto K, Nakayama M, Ichimura K, Onozaki T, Yamaguchi H. Sequence analysis of the genome of carnation (*Dianthus caryophyllus* L.). *DNA Res.* 2014 Jun 1;21(3):231-41.
7. Sunil M, Hariharan AK, Nayak S, Gupta S, Nambisan SR, Gupta RP, Panda B, Choudhary B, Srinivasan S. The draft genome and transcriptome of *Amaranthus hypochondriacus*: a C4 dicot producing high-lysine edible pseudo-cereal. *DNA Res.* 2014 Dec 1;21(6):585-602.
8. Cai J, Liu X, Vanneste K, Proost S, Tsai WC, Liu KW, Chen LJ, He Y, Xu Q, Bian C, Zheng Z. The genome sequence of the orchid *Phalaenopsis equestris*. *Nat. Genet.* 2015 Jan;47(1):65-72.
9. Yan L, Wang X, Liu H, Tian Y, Lian J, Yang R, Hao S, Wang X, Yang S, Li Q, Qi S. The genome of *Dendrobium officinale* illuminates the biology of the important traditional Chinese orchid herb. *Mol. Plant.* 2015 Jun 1;8(6):922-34.
10. MD, Russo G, Schlapbach R, Huu CN, Lenhard M, Conti E. The draft genome of *Primula veris* yields insights into the molecular basis of heterostyly. *Genome Biol.* 2015 Dec;16(1):1-7.
11. De Vega JJ, Ayling S, Hegarty M, Kudrna D, Goicoechea JL, Ergon Å, Rognli OA, Jones C, Swain M, Geurts R, Lang C. Red clover (*Trifolium pratense* L.) draft genome provides a platform for trait improvement. *Sci. Rep.* 2015 Nov 30;5(1):17394.
12. Byrne SL, Nagy I, Pfeifer M, Armstead I, Swain S, Studer B, Mayer K, Campbell JD, Czaban A, Hentrup S, Panitz F. A synteny‐based draft genome sequence of the forage grass *Lolium perenne*. *Plant Journal.* 2015 Nov;84(4):816-26.
13. Kellner F, Kim J, Clavijo BJ, Hamilton JP, Childs KL, Vaillancourt B, Cepela J, Habermann M, Steuernagel B, Clissold L, McLay K. Genome‐guided investigation of plant natural product biosynthesis. *Plant Journal.* 2015 May;82(4):680-92.
14. Xiao L, Yang G, Zhang L, Yang X, Zhao S, Ji Z, Zhou Q, Hu M, Wang Y, Chen M, Xu Y. The resurrection genome of *Boea hygrometrica*: A blueprint for survival of dehydration. *Proc. Natl. Acad. Sci. U.S.A.* 2015 May 5;112(18):5833-7.
15. Zhang GQ, Xu Q, Bian C, Tsai WC, Yeh CM, Liu KW, Yoshida K, Zhang LS, Chang SB, Chen F, Shi Y. The *Dendrobium catenatum* Lindl. genome sequence provides insights into polysaccharide synthase, floral development and adaptive evolution. Sci. Rep. 2016 Jan 12;6(1):19029.
16. Hoshino A, Jayakumar V, Nitasaka E, Toyoda A, Noguchi H, Itoh T, Shin-i T, Minakuchi Y, Koda Y, Nagano AJ, Yasugi M. Genome sequence and analysis of the Japanese morning glory Ipomoea nil. Nat. Commun. 2016 Nov 8;7(1):13295.
17. Kim YM, Kim S, Koo N, Shin AY, Yeom SI, Seo E, Park SJ, Kang WH, Kim MS, Park J, Jang I. Genome analysis of *Hibiscus syriacus* provides insights of polyploidization and indeterminate flowering in woody plants. DNA Res. 2017 Feb 1;24(1):71-80.
18. Bombarely A, Moser M, Amrad A, Bao M, Bapaume L, Barry CS, Bliek M, Boersma MR, Borghi L, Bruggmann R, Bucher M. Insight into the evolution of the Solanaceae from the parental genomes of *Petunia hybrida*. Nat. Plants. 2016 May 27;2(6):1-9.
19. Lu M, An H, Li L. Genome survey sequencing for the characterization of the genetic background of *Rosa roxburghii* Tratt and leaf ascorbate metabolism genes. PLoS One. 2016 Feb 5;11(2):e0147530.
20. Tanaka H, Hirakawa H, Kosugi S, Nakayama S, Ono A, Watanabe A, Hashiguchi M, Gondo T, Ishigaki G, Muguerza M, Shimizu K. Sequencing and comparative analyses of the genomes of zoysiagrasses. DNA Res. 2016 Apr 1;23(2):171-80.
21. Huang JZ, Lin CP, Cheng TC, Huang YW, Tsai YJ, Cheng SY, Chen YW, Lee CP, Chung WC, Chang BC, Chin SW. The genome and transcriptome of Phalaenopsis yield insights into floral organ development and flowering regulation. *PeerJ*. 2016 May 12;4:e2017.
22. Zhang GQ, Liu KW, Li Z, Lohaus R, Hsiao YY, Niu SC, Wang JY, Lin YC, Xu Q, Chen LJ, Yoshida K. The Apostasia genome and the evolution of orchids. *Nature.* 2017 Sep 21;549(7672):379-83.
23. Yang X, Hu R, Yin H, Jenkins J, Shu S, Tang H, Liu D, Weighill DA, Cheol Yim W, Ha J, Heyduk K. The Kalanchoë genome provides insights into convergent evolution and building blocks of crassulacean acid metabolism. *Nature commun.* 2017 Dec 1;8(1):1899.
24. Badouin H, Gouzy J, Grassa CJ, Murat F, Staton SE, Cottret L, Lelandais-Brière C, Owens GL, Carrère S, Mayjonade B, Legrand L. The sunflower genome provides insights into oil metabolism, flowering and Asterid evolution. *Nature.* 2017 Jun 1;546(7656):148-52.
25. Zhang L, Xu P, Cai Y, Ma L, Li S, Li S, Xie W, Song J, Peng L, Yan H, Zou L. The draft genome assembly of *Rhododendron delavayi* Franch. var. delavayi. *GigaScience.* 2017 Oct;6(10):gix076.
26. Fu Y, Li L, Hao S, Guan R, Fan G, Shi C, Wan H, Chen W, Zhang H, Liu G, Wang J. Draft genome sequence of the Tibetan medicinal herb Rhodiola crenulata. *Gigascience.* 2017 Jun;6(6):gix033.
27. Zhao D, Hamilton JP, Pham GM, Crisovan E, Wiegert-Rininger K, Vaillancourt B, DellaPenna D, Buell CR. De novo genome assembly of Camptotheca acuminata, a natural source of the anti-cancer compound camptothecin. *Gigascience.* 6: 1–7.
28. Nakamura N, Hirakawa H, Sato S, Otagaki S, Matsumoto S, Tabata S, Tanaka Y. Genome structure of *Rosa multiflora*, a wild ancestor of cultivated roses. *DNA Res.* 2018 Apr 1;25(2):113-21.
29. Saint-Oyant LH, Ruttink T, Hamama L, Kirov I, Lakhwani D, Zhou NN, Bourke PM, Daccord N, Leus L, Schulz D, Van de Geest H. A high-quality genome sequence of *Rosa chinensis* to elucidate ornamental traits. *Nat. Plants.* 4: 473–484.
30. Chao YT, Chen WC, Chen CY, Ho HY, Yeh CH, Kuo YT, Su CL, Yen SH, Hsueh HY, Yeh JH, Hsu HL. Chromosome‐level assembly, genetic and physical mapping of *Phalaenopsis aphrodite* genome provides new insights into species adaptation and resources for orchid breeding. *Plant Biotechnol. J.* 2018 Dec;16(12):2027-41.
31. Song C, Liu Y, Song A, Dong G, Zhao H, Sun W, Ramakrishnan S, Wang Y, Wang S, Li T, Niu Y. The *Chrysanthemum nankingense* genome provides insights into the evolution and diversification of chrysanthemum flowers and medicinal traits. *Mol Plant.* 2018 Dec 3;11(12):1482-91.
32. Dong AX, Xin HB, Li ZJ, Liu H, Sun YQ, Nie S, Zhao ZN, Cui RF, Zhang RG, Yun QZ, Wang XN. High-quality assembly of the reference genome for scarlet sage, *Salvia splendens*, an economically important ornamental plant. GigaScience. 2018 Jul;7(7):giy068.
33. Cocker JM, Wright J, Li J, Swarbreck D, Dyer S, Caccamo M, Gilmartin PM. *Primula vulgaris* (primrose) genome assembly, annotation and gene expression, with comparative genomics on the heterostyly supergene. *Sci. Rep.* 2018 Dec 18;8(1):17942.
34. Griesmann M, Chang Y, Liu X, Song Y, Haberer G, Crook MB, Billault-Penneteau B, Lauressergues D, Keller J, Imanishi L, Roswanjaya YP. Phylogenomics reveals multiple losses of nitrogen-fixing root nodule symbiosis. *Science.* 2018 Jul 13;361(6398):eaat1743.
35. Zhang L, Chen F, Zhang X, Li Z, Zhao Y, Lohaus R, Chang X, Dong W, Ho SY, Liu X, Song A. The water lily genome and the early evolution of flowering plants. *Nature.* 2020 Jan 2;577(7788):79-84.
36. Li SF, Wang J, Dong R, Zhu HW, Lan LN, Zhang YL, Li N, Deng CL, Gao WJ. Chromosome-level genome assembly, annotation and evolutionary analysis of the ornamental plant Asparagus setaceus. *Hortic. Res.* 2020 Dec 1;7.
37. Yamashiro T, Shiraishi A, Satake H, Nakayama K. Draft genome of *Tanacetum cinerariifolium*, the natural source of mosquito coil. *Sci. Rep.* 2019 Dec 3;9(1):18249.
38. Hirakawa H, Sumitomo K, Hisamatsu T, Nagano S, Shirasawa K, Higuchi Y, Kusaba M, Koshioka M, Nakano Y, Yagi M, Yamaguchi H. De novo whole-genome assembly in *Chrysanthemum seticuspe*, a model species of Chrysanthemums, and its application to genetic and gene discovery analysis. *DNA Res.* 2019 Jun 1;26(3):195-203.
39. Li M, Zhang D, Gao Q, Luo Y, Zhang H, Ma B, Chen C, Whibley A, Zhang YE, Cao Y, Li Q. Genome structure and evolution of *Antirrhinum majus* L. *Nat. Plants*. 2019 Feb;5(2):174-83.
40. Palfalvi G, Hackl T, Terhoeven N, Shibata TF, Nishiyama T, Ankenbrand M, Becker D, Förster F, Freund M, Iosip A, Kreuzer I. Genomes of the Venus flytrap and close relatives unveil the roots of plant carnivory. *Curr. Biol.* 2020 Jun 22;30(12):2312-20.
41. Lv S, Cheng S, Wang Z, Li S, Jin X, Lan L, Yang B, Yu K, Ni X, Li N, Hou X. Draft genome of the famous ornamental plant *Paeonia suffruticosa*. *Ecol. Evol.* 2020 Jun;10(11):4518-30.
42. Soza VL, Lindsley D, Waalkes A, Ramage E, Patwardhan RP, Burton JN, Adey A, Kumar A, Qiu R, Shendure J, Hall B. The Rhododendron genome and chromosomal organization provide insight into shared whole-genome duplications across the heath family (Ericaceae). Genome Biol. Evol*.* 2019 Dec;11(12):3353-71.
43. Palfalvi G, Hackl T, Terhoeven N, Shibata TF, Nishiyama T, Ankenbrand M, Becker D, Förster F, Freund M, Iosip A, Kreuzer I. Genomes of the Venus flytrap and close relatives unveil the roots of plant carnivory. *Curr. Biol.* 2020 Jun 22;30(12):2312-20.
44. Lv Q, Qiu J, Liu J, Li Z, Zhang W, Wang Q, Fang J, Pan J, Chen Z, Cheng W, Barker MS. The *Chimonanthus salicifolius* genome provides insight into magnoliid evolution and flavonoid biosynthesis. *Plant Journal.* 2020 Aug;103(5):1910-23.
45. Liu G, Sun M, Zou P, Zhang W, Ni J. The complete chloroplast genome sequence of a popular ornamental plant *Calibrachoa hybrida* (Solanaceae: Petunioideae). *Mitochondrial DNA Part B.* 2020 Jul 2;5(3):3374-5.
46. Shang J, Tian J, Cheng H, Yan Q, Li L, Jamal A, Xu Z, Xiang L, Saski CA, Jin S, Zhao K. The chromosome-level wintersweet (*Chimonanthus praecox*) genome provides insights into floral scent biosynthesis and flowering in winter. *Genome Biol.* 2020 Dec;21(1):1-28.
47. Nakano M, Hirakawa H, Fukai E, Toyoda A, Kajitani R, Minakuchi Y, Itoh T, Higuchi Y, Kozuka T, Bono H, Shirasawa K. A chromosome-level genome sequence of *Chrysanthemum seticuspe*, a model species for hexaploid cultivated chrysanthemum.*Commun. Biol.* 2021 Oct 7;4(1):1167.
48. Hongmei S, Wenrui H, Dianyun H, Yang X. Complete chloroplast genome sequence of *Dendranthema zawadskii* Herbich. *Mitochondrial DNA Part B.* 2021 Aug 3;6(8):2117-9.
49. Wang X, Gao Y, Wu X, Wen X, Li D, Zhou H, Li Z, Liu B, Wei J, Chen F, Chen F. High‐quality evergreen azalea genome reveals tandem duplication‐facilitated low‐altitude adaptability and floral scent evolution. *Plant Biotechnol. J.* 2021 Dec;19(12):2544-60.
50. Wen X, Li J, Wang L, Lu C, Gao Q, Xu P, Pu Y, Zhang Q, Hong Y, Hong L, Huang H. The *Chrysanthemum lavandulifolium* genome and the molecular mechanism underlying diverse capitulum types. *Hortic. Res.* 2022;9:uhab022.
51. van Lieshout N, van Kaauwen M, Kodde L, Arens P, Smulders MJ, Visser RG, Finkers R. De novo whole-genome assembly of *Chrysanthemum makinoi*, a key wild chrysanthemum. *G3.* 2022 Jan 1;12(1):jkab358.
52. Liang Y, Li F, Gao Q, Jin C, Dong L, Wang Q, Xu M, Sun F, Bi B, Zhao P, Li S. The genome of Eustoma grandiflorum reveals the whole‐genome triplication event contributing to ornamental traits in cultivated lisianthus. *Plant Biotechnol. J.* 2022 Oct;20(10):1856.
53. Song A, Su J, Wang H, Zhang Z, Zhang X, Van de Peer Y, Chen F, Fang W, Guan Z, Zhang F, Wang Z. Analyses of a chromosome-scale genome assembly reveal the origin and evolution of cultivated chrysanthemum. *Nat. Commun.* 2023 Apr 11;14(1):2021.

**References of Fig. 2:** List of candidate genes involved in the regulation or improvement of important traits in different ornamental plant species (cut flower, loose flower, bedding plants, flowering house plants, and foliage house plants). The superscript represent the relevant citation for a associated gene. The citations are provided in the supplementary file **(see references S54-S289)**.

1. Ito H, Ochiai M, Kato H, Shiratake K, Takemoto D, Otagaki S, Matsumoto S. Rose phytoene desaturase gene silencing by apple latent spherical virus vectors. *HortScience.* 2012 Sep 1;47(9):1278-82.
2. Katsumoto Y, Fukuchi-Mizutani M, Fukui Y, Brugliera F, Holton TA, Karan M, Nakamura N, Yonekura-Sakakibara K, Togami J, Pigeaire A, Tao GQ. Engineering of the rose flavonoid biosynthetic pathway successfully generated blue-hued flowers accumulating delphinidin. *Plant Cell Physiol.* 2007 Nov 1;48(11):1589-600.
3. Nakamura N, Katsumoto Y, Brugliera F, Demelis L, Nakajima D, Suzuki H, Tanaka Y. Flower color modification in *Rosa hybrida* by expressing the S-adenosylmethionine: anthocyanin 3′, 5′-O-methyltransferase gene from *Torenia hybrida*. *Plant Biotechnol.* 2015 Jun 25;32(2):109-17.
4. Xu J, Shin JY, Park PM, An HR, Kim YJ, Kim SJ, Lee SY. Flower color modification through co-overexpression of the VtF3′ 5′ H and RhNHX genes in *Rosa hybrida*. *Plant Cell, Tissue Organ Cult.* 2023 May;153(2):403-16.
5. Zhang Y, Yu D, Cheng Y, Tang H, Wang Q, Zhang Y. Cloning and Expression Analysis of Chalcone Synthase Gene from Rosa hybrida. *Biotechnol. Bulletin*. 2013 Jul 19(7):66.
6. Luo P, Ning G, Wang Z, Shen Y, Jin H, Li P, Huang S, Zhao J, Bao M. Disequilibrium of flavonol synthase and dihydroflavonol-4-reductase expression associated tightly to white vs. red color flower formation in plants. *Front. Plant Sci.* 2016 Jan 13;6:1257.
7. Li Z, Zhao M, Jin J, Zhao L, Xu Z. Anthocyanins and their biosynthetic genes in three novel-colored Rosa rugosa cultivars and their parents. *Plant Physiol. Biochem*. 2018 Aug 1;129:421-8.
8. He G, Zhang R, Jiang S, Wang H, Ming F. The MYB transcription factor *RcMYB1* plays a central role in rose anthocyanin biosynthesis. *Hortic. Res.* 2023: 10(6):uhad080.
9. Zvi MM, Shklarman E, Masci T, Kalev H, Debener T, Shafir S, Ovadis M, Vainstein A. *PAP1* transcription factor enhances production of phenylpropanoid and terpenoid scent compounds in rose flowers. *New Phytol.* 2012 Jul;195(2):335-45.
10. Zhou Lj, Huang Rh, Liu Th, Liu Wc, Chen Yy, Lyu Pf, Le Lu, Pan Ht, Chao Yu, Zhang Qx. Volatile metabolome and transcriptome reveal fragrance release rhythm and molecular mechanisms of *Rosa yangii*. *J Integr Agric.* 2023 Jun 15.
11. Raymond O, Gouzy J, Just J, Badouin H, Verdenaud M, Lemainque A, Vergne P, Moja S, Choisne N, Pont C, Carrere S. The Rosa genome provides new insights into the domestication of modern roses. *Nat. Gen.* 2018 Jun;50(6):772-7.
12. Hirata H, Ohnishi T, Watanabe N. Biosynthesis of floral scent 2-phenylethanol in rose flowers. *Biosci. Biotechnol. Biochem*. 2016 Oct 2;80(10):1865-73.
13. Zakizadeh H, Lütken H, Sriskandarajah S, Serek M, Müller R. Transformation of miniature potted rose (*Rosa hybrida* cv. Linda) with P SAG12-ipt gene delays leaf senescence and enhances resistance to exogenous ethylene. *Plant cell Rep.* 2013 Feb;32:195-205.
14. Wang C, Li Y, Wang N, Yu Q, Li Y, Gao J, Zhou X, Ma N. An efficient CRISPR/Cas9 platform for targeted genome editing in rose (*Rosa hybrida*). Journal of Integrative Plant Biology. 2023 Apr;65(4):895-9.
15. Chen JR, Lü JJ, Liu R, Xiong XY, Wang TX, Chen SY, Guo LB, Wang HF. DREB1C from Medicago truncatula enhances freezing tolerance in transgenic *M. truncatula* and China Rose (*Rosa chinensis* Jacq.). *Plant Growth Regul.* 2010 Apr;60:199-211.
16. Jiang C, Bi Y, Zhang R, Feng S. Expression of *RcHSP70*, heat shock protein 70 gene from Chinese rose, enhances host resistance to abiotic stresses. *Sci.* *Rep*. 2020 Feb 12;10(1):2445.
17. Gangwar H, Kumari P, Gahlaut V, Kumar S, Jaiswal V. Identification and comprehensive analysis of MIPSs in Rosaceae and their expression under abiotic stresses in rose (*Rosa chinensis*). *Front. Plant Sci.* 2022 Nov 3;13:1021297.
18. Marchant R, Davey MR, Lucas JA, Lamb CJ, Dixon RA, Power JB. Expression of a chitinase transgene in rose (Rosa hybrida L.) reduces development of blackspot disease *(Diplocarpon rosae* Wolf). *Mol. Breed.* 1998 Jun;4:187-94.
19. Martens S, Forkmann G. Cloning and expression of flavone synthase II from Gerbera hybrids. *Plant Journal*. 1999 Dec;20(5):611-8.
20. Helariutta Y, Elomaa P, Kotilainen M, Seppänen P, Teeri TH. Cloning of cDNA coding for dihydroflavonol-4-reductase (*DFR*) and characterization of dfr expression in the corollas of *Gerbera hybrida* var. Regina (Compositae). *Plant Mol. Biol.* 1993 May;22:183-93.
21. Deng X, Bashandy H, Ainasoja M, Kontturi J, Pietiäinen M, Laitinen RA, Albert VA, Valkonen JP, Elomaa P, Teeri TH. Functional diversification of duplicated chalcone synthase genes in anthocyanin biosynthesis of *Gerbera hybrida*. *New Phyto*l. 2014 Mar;201(4):1469-83.
22. Zhong C, Tang Y, Pang B, Li X, Yang Y, Deng J, Feng C, Li L, Ren G, Wang Y, Peng J. The R2R3-MYB transcription factor *GhMYB1a* regulates flavonol and anthocyanin accumulation in *Gerbera hybrida*. *Hortic. Res.* 2020 Dec 1;7.
23. Helariutta Y, Elomaa P, Kotilainen M, Seppänen P, Teeri TH. Cloning of cDNA coding for dihydroflavonol-4-reductase (DFR) and characterization of *dfr* expression in the corollas of *Gerbera hybrida* var. Regina (Compositae). *Plant Mol. Biol.* 1993 May;22:183-93.
24. Hamedan HJ, Sohani MM, Aalami A, Nazarideljou MJ. Genetic engineering of lignin biosynthesis pathway improved stem bending disorder in cut gerbera (*Gerbera jamesonii*) flowers. *Sci. Hort.* 2019 ;245:274-9.
25. Korbin M, Podwyszynska M, Komorowska B, Wawrzynczak D. Transformation of Gerbera plants with Tomato spotted wilt virus (TSWV) nucleoprotein gene. *In XX International Eucarpia Symposium, Section Ornamentals, Strategies for New Ornamentals-Part II.* 572 2001 Jul 3;149-157.
26. Bhattarai K, Conesa A, Xiao S, Peres NA, Clark DG, Parajuli S, Deng Z. Sequencing and analysis of gerbera daisy leaf transcriptomes reveal disease resistance and susceptibility genes differentially expressed and associated with powdery mildew resistance. *BMC Plant Biol.* 2020 Dec;20(1):1-7.
27. Gautam KK, Raj R, Kumar S, Agrawal L, Chauhan PS, Raj SK. Development of transgenic cucumber mosaic virus (CMV) resistant gerbera plants expressing CMV coat protein gene. *Indian J Exp Biol.* 2022 Jan 31;60(02):121-30.
28. Ruokolainen S, Ng YP, Broholm SK, Albert VA, Elomaa P, Teeri TH. Characterization of SQUAMOSA-like genes in *Gerbera hybrida*, including one involved in reproductive transition. *BMC Plant Biol.* 2010 Dec;10:1-1.
29. Ren G, Li L, Patra B, Li N, Zhou Y, Zhong C, Wang Y, Yuan L, Wang X. *GhTCP7* suppresses petal expansion by interacting with the WIP-type zinc finger protein *GhWIP2* in *Gerbera hybrida*. *J. Exp. Bot.* 2023:erad152.
30. Zhao Y, Broholm SK, Wang F, Rijpkema AS, Lan T, Albert VA, Teeri TH, Elomaa P. TCP and MADS-box transcription factor networks regulate heteromorphic flower type identity in *Gerbera hybrida*. *Plant Physiol.* 2020 Nov 1;184(3):1455-68.
31. Ruokolainen S, Ng YP, Albert VA, Elomaa P, Teeri TH. Over-expression of the Gerbera hybrida At-SOC1-like1 gene Gh-SOC1 leads to floral organ identity deterioration. Annals of Botany. 2011 Jun 1;107(9):1491-9.
32. Zhang T, Zhao Y, Juntheikki I, Mouhu K, Broholm SK, Rijpkema AS, Kins L, Lan T, Albert VA, Teeri TH, Elomaa P. Dissecting functions of SEPALLATA‐like MADS box genes in patterning of the pseudanthial inflorescence of *Gerbera hybrida*. *New Phytol.* 2017 Nov;216(3):939-54.
33. Ozeki Y, Iijima L, Higuchi K, Miyahara T, Sasaki N, Tsujimoto T, Abe Y, Matsuba Y, Nishizaki Y, Suzuki-Wagner A, Ogata J. Molecular mechanisms of carnation flower colors via anthocyanin and flavonoid biosynthetic pathways. *Carnation Genome.* 2020:99-117.
34. Totsuka A, Okamoto E, Miyahara T, Kouno T, Cano EA, Sasaki N, Watanabe A, Tasaki K, Nishihara M, Ozeki Y. Repressed expression of a gene for a basic helix-loop-helix protein causes a white flower phenotype in carnation. *Breed. Sci.* 2018;68(1):139-43.
35. Fukui Y, Tanaka Y, Kusumi T, Iwashita T, Nomoto K. A rationale for the shift in colour towards blue in transgenic carnation flowers expressing the flavonoid 3′, 5′-hydroxylase gene. *Phytochem.* 2003 May 1;63(1):15-23.
36. Lavy M, Zuker A, Lewinsohn E, Larkov O, Ravid U, Vainstein A, Weiss D. Linalool and linalool oxide production in transgenic carnation flowers expressing the *Clarkia breweri linalool synthase* gene. *Mol. Breed.* 2002 Jun;9:103-11.
37. Zhang X, Lin S, Peng D, Wu Q, Liao X, Xiang K, Wang Z, Tembrock LR, Bendahmane M, Bao M, Wu Z. Integrated multi‐omic data and analyses reveal the pathways underlying key ornamental traits in carnation flowers. *Plant Biotechnol. J.* 2022 Jun;20(6):1182-96.
38. Xu H, Luo D, Zhang F. *DcWRKY75* promotes ethylene induced petal senescence in carnation (*Dianthus caryophyllus* L.). *Plant Journal.* 2021 Dec;108(5):1473-92.
39. Xu H, Wang S, Larkin RM, Zhang F. The transcription factors DcHB30 and DcWRKY75 antagonistically regulate ethylene-induced petal senescence in carnation (*Dianthus caryophyllus*). *J. Exp. Bot.* 2022 Dec 8;73(22):7326-43.
40. Wang T, Sun Z, Wang S, Feng S, Wang R, Zhu C, Zhong L, Cheng Y, Bao M, Zhang F. DcWRKY33 promotes petal senescence in carnation (*Dianthus caryophyllus* L.) by activating genes involved in the biosynthesis of ethylene and abscisic acid and accumulation of reactive oxygen species. *Plant Journal*. 2023 Feb;113(4):698-715.
41. Li W, Wan XL, Yu JY, Wang KL, Zhang J. Genome-wide identification, classification, and expression analysis of the Hsf gene family in carnation (*Dianthus caryophyllus*). *Int. J. Mol. Sci.* 2019 Oct 22;20(20):5233.
42. Sun Y, Hu D, Xue P, Wan X. Identification of the DcHsp20 gene family in carnation (*Dianthus caryophyllus*) and functional characterization of *DcHsp17*. 8 in heat tolerance. *Planta.* 2022 Jul;256(1):2.
43. Shirasawa-Seo N, Nakamura S, Ukai N, Honkura R, Iwai T, Ohashi Y. Ectopic expression of an oat thionin gene in carnation plants confers enhanced resistance to bacterial wilt disease. *Plant Biotechnol.* 2002;19(5):311-7.
44. Ahn BJ, Shin HY, Hwang KH, Min BH, Joung HY. Transformation of carnations with jasmonate methyl transferase gene for fusarium tolerance. *In Vitro Cell. Dev. Biol.* 2004 Apr 1;40:45A.
45. Casanova E, Zuker A, Trillas MI, Moysset L, Vainstein A. The *rolC* gene in carnation exhibits cytokinin-and auxin-like activities. *Sci. Hort.* 2003 Feb 17;97(3-4):321-31.
46. Meng LS, Song JP, Sun SB, Wang CY. The ectopic expression of *PttKN1* gene causes pleiotropic alternation of morphology in transgenic carnation (Dianthus caryophyllus L.). *Acta Physiol. Plant.* 2009 Nov;31:1155-64.
47. Yamagishi M, Shimoyamada Y, Nakatsuka T, Masuda K. Two *R2R3-MYB* genes, homologs of petunia AN2, regulate anthocyanin biosyntheses in flower tepals, tepal spots and leaves of Asiatic hybrid lily. *Plant Cell Physiol.* 2010 Mar 1;51(3):463-74.
48. Sakai M, Yamagishi M, Matsuyama K. Repression of anthocyanin biosynthesis by R3-MYB transcription factors in lily (Lilium spp.). *Plant cell Rep.* 2019 May 1;38:609-22.
49. Fang S, Lin M, Ali MM, Zheng Y, Yi X, Wang S, Chen F, Lin Z. *LhANS-rr1, LhDFR*, and *LhMYB114* Regulate Anthocyanin Biosynthesis in Flower Buds of Lilium ‘Siberia’. *Genes*. 2023 Feb 23;14(3):559.
50. Lai YS, Shimoyamada Y, Nakayama M, Yamagishi M. Pigment accumulation and transcription of *LhMYB12* and anthocyanin biosynthesis genes during flower development in the Asiatic hybrid lily (Lilium spp.). *Plant Sci.* 2012 Sep 1;193:136-47.
51. Yoshida K, Oyama-Okubo N, Yamagishi M. An R2R3-MYB transcription factor *ODORANT1* regulates fragrance biosynthesis in lilies (Lilium spp.). *Mol. Breed.* 2018 Dec;38:1-4.
52. Zhang T, Guo Y, Shi X, Yang Y, Chen J, Zhang Q, Sun M. Overexpression of LiTPS2 from a cultivar of lily (Lilium ‘Siberia’) enhances the monoterpenoids content in tobacco flowers. *Plant Physiol. Biochem.* 2020 Jun 1;151:391-9.
53. Feng Y, Guo Z, Zhong J, Liang Y, Zhang P, Sun M. The LibHLH22 and LibHLH63 from Lilium ‘Siberia’Can Positively Regulate Volatile Terpenoid Biosynthesis. *Horticulturae.* 2023;9(4):459.
54. Zeng Z, Lyu T, Lyu Y. *LoSWEET14,* a Sugar Transporter in Lily, Is Regulated by Transcription Factor *LoABF2* to Participate in the ABA Signaling Pathway and Enhance Tolerance to Multiple Abiotic Stresses in Tobacco. I*nt. J. Mol. Sci*. 2022 Dec 1;23(23):15093.
55. Wang L, Guo Z, Zhang Y, Wang Y, Yang G, Yang L, Wang R, Xie Z. Characterization of LhSorP5CS, a gene catalyzing proline synthesis in Oriental hybrid lily Sorbonne: molecular modelling and expression analysis. *Bot. Stud.* 2017 Dec;58:1-8.
56. Wang C, Zhou Y, Yang X, Zhang B, Xu F, Wang Y, Song C, Yi M, Ma N, Zhou X, He J. The heat stress transcription factor *LlHsfA4* enhanced basic thermotolerance through regulating ROS metabolism in lilies (*Lilium longiflorum*). *Int. J. Mol. Sci.* 2022 Jan 5;23(1):572.
57. Wu Z, Liang J, Zhang S, Zhang B, Zhao Q, Li G, Yang X, Wang C, He J, Yi M. A canonical DREB2-type transcription factor in lily is post-translationally regulated and mediates heat stress response. *Front. Plant Sci.* 2018 Mar 8;9:243.
58. Núñez de Cáceres González FF, Davey MR, Cancho Sanchez E, Wilson ZA. Conferred resistance to *Botrytis cinerea* in Lilium by overexpression of the RCH10 chitinase gene. *Plant Cell Rep.* 2015 Jul;34:1201-9.
59. Kumari S, Kanth BK, Ahn JY, Kim JH, Lee GJ. Genome-wide transcriptomic identification and functional insight of lily *WRKY* genes responding to botrytis fungal disease. *Plants.* 2021 Apr 15;10(4):776.
60. Wang L, Guo Z, Zhang Y, Wang Y, Yang G, Yang L, Wang L, Wang R, Xie Z. Overexpression of LhSorNPR1, a NPR1-like gene from the oriental hybrid lily ‘Sorbonne’, conferred enhanced resistance to *Pseudomonas syringae* pv. tomato DC3000 in Arabidopsis*. Physiol. Mol. Biol. Plants.* 2017 Oct;23:793-808.
61. Azadi P, Otang NV, Supaporn H, Khan RS, Chin DP, Nakamura I, Mii M. Increased resistance to cucumber mosaic virus (CMV) in Lilium transformed with a defective *CMV* replicase gene. *Biotechnol. Lett.* 2011 Jun;33:1249-55.
62. Tzeng TY, Chen HY, Yang CH. Ectopic expression of carpel-specific MADS box genes from lily and lisianthus causes similar homeotic conversion of sepal and petal in Arabidopsis. *Plant Physiol*. 2002 Dec 1;130(4):1827-36.
63. Chen MK, Lin IC, Yang CH. Functional analysis of three lily (*Lilium longiflorum*) APETALA1-like MADS box genes in regulating floral transition and formation. *Plant cell Physiol.* 2008 May 1;49(5):704-17.
64. Noda N, Kanno Y, Kato N, Kazuma K, Suzuki M. Regulation of gene expression involved in flavonol and anthocyanin biosynthesis during petal development in lisianthus (Eustoma grandiflorum). *Physiol. Plant.* 2004 Nov;122(3):305-13.
65. Aranovich D, Lewinsohn E, Zaccai M. Post-harvest enhancement of aroma in transgenic lisianthus (Eustoma grandiflorum) using the Clarkia breweri benzyl alcohol acetyltransferase (BEAT) gene. *Postharvest Biol. Technol*. 2007 Feb 1;43(2):255-60.
66. Wang, L., Xue, W., Li, X., Li, J., Wu, J., Xie, L., Kawabata, S., Li, Y. and Zhang, Y., 2020. EgMIXTA1, a MYB-type transcription factor, promotes cuticular wax formation in *Eustoma grandiflorum* leaves. *Frontiers in Plant Science*, *11*, p.524947.
67. Nakano Y, Kawashima H, Kinoshita T, Yoshikawa H, Hisamatsu T. Characterization of FLC, SOC1 and FT homologs in *Eustoma grandiflorum*: effects of vernalization and post‐vernalization conditions on flowering and gene expression. *Physiol. Plant.* 2011 Apr;141(4):383-93.
68. Mino M, Oka M, Tasaka Y, Iwabuchi M. Thermoinduction of genes encoding the enzymes of gibberellin biosynthesis and a putative negative regulator of gibberellin signal transduction in *Eustoma grandiflorum*. *Plant cell Rep.* 2003 Sep;22:159-65.
69. Zhang C, Wang Y, Wang W, Cao Z, Fu Q, Bao M, He Y. Functional analysis of the marigold (*Tagetes erecta*) lycopene ε-cyclase (TeLCYe) promoter in transgenic tobacco. *Mol. Biotechnol*. 2019 Sep 1;61:703-13.
70. Zhang C, Wang N, Wang N, Bao M, He Y. Cloning and functional analysis of lycopene β-cyclase promoter of marigold (*Tagetes erecta*). *Sci. Agric. Sin.* 2017;50(24):4779-89.
71. Zhang C, Sun Y, Yu X, Li H, Bao M, He Y. Functional conservation and divergence of five AP1/FUL-like genes in marigold (*Tagetes erecta* L.). *Genes.* 2021 Dec 17;12(12):2011
72. Zhang C, Wei L, Yu X, Li H, Wang W, Wu S, Duan F, Bao M, Chan Z, He Y. Functional conservation and divergence of SEPALLATA-like genes in the development of two-type florets in marigold. *Plant Sci*. 2021 Aug 1;309:110938.
73. He YH, Ning GG, Sun YL, Qi YC, Bao MZ. Identification of a SCAR marker linked to a recessive male sterile gene (Tems) and its application in breeding of marigold (*Tagetes erecta*). *Plant Breed.* 2009 Feb;128(1):92-6.
74. Liu J, Wang Y, Zhang M, Wang Y, Deng X, Sun H, Yang D, Xu L, Song H, Yang M. Color fading in lotus (*Nelumbo nucifera*) petals is manipulated both by anthocyanin biosynthesis reduction and active degradation. *Plant Physiol. Biochem.* 2022 May 15;179:100-7.
75. Zhu HH, Yang JX, Xiao CH, Mao TY, Zhang J, Zhang HY. Differences in flavonoid pathway metabolites and transcripts affect yellow petal colouration in the aquatic plant *Nelumbo nucifera*. *BMC Plant Biol.* 2019 Dec;19(1):1-8.
76. Liu J, Wang Y, Deng X, Zhang M, Sun H, Gao L, Song H, Xin J, Ming R, Yang D, Yang M. Transcription factor NnMYB5 controls petal color by regulating GLUTATHIONE S-TRANSFERASE2 in *Nelumbo nucifera*. *Plant Physiol*. 2023 Jun 22:kiad363.
77. Zheng P, Sun H, Liu J, Lin J, Zhang X, Qin Y, Zhang W, Xu X, Deng X, Yang D, Wang M. Comparative analyses of American and Asian lotus genomes reveal insights into petal color, carpel thermogenesis and domestication. *Plant Journal*. 2022 Jun;110(5):1498-515.
78. Wu Y, Wu S, Shi Y, Jiang L, Yang J, Wang X, Zhu K, Zhang H, Zhang J. Integrated metabolite profiling and transcriptome analysis reveal candidate genes involved in the formation of yellow *Nelumbo nucifera*. *Genomics*. 2022 Nov 1;114(6):110513.
79. Suzuki S, Nishihara M, Nakatsuka T, Misawa N, Ogiwara I, Yamamura S. Flower color alteration in Lotus japonicus by modification of the carotenoid biosynthetic pathway. *Plant cell Rep.* 2007 Jul;26:951-9.
80. Gao Z, Yang X, Chen J, Rausher MD, Shi T. Expression inheritance and constraints on cis-and trans-regulatory mutations underlying lotus color variation. *Plant Physiol* 2023 Mar 1;191(3):1662-83.
81. Robbins MP, Paolocci F, Hughes JW, Turchetti V, Allison G, Arcioni S, Morris P, Damiani F. Sn, a maize bHLH gene, modulates anthocyanin and condensed tannin pathways in Lotus corniculatus. *J. Exp. Bot.* 2003 Jan 2;54(381):239-48.
82. Liu R, Shi H, Wang Y, Chen S, Deng J, Liu Y, Li S, Chan Z. Comparative physiological analysis of lotus (*Nelumbo nucifer*a) cultivars in response to salt stress and cloning of NnCIPK genes. *Sci. Hort.* 2014 Jun 27;173:29-36.
83. Zhao S, Jiang T, Zhang Y, Zhang K, Feng K, Wu P, Li L. Identification of the NAC Transcription factors and their function in ABA and salinity response in *Nelumbo nucifera*. *Int. J. Mol. Sci.* 2022 Oct 16;23(20):12394.
84. Zhou Y, Chen H, Chu P, Li Y, Tan B, Ding Y, Tsang EW, Jiang L, Wu K, Huang S. NnHSP17. 5, a cytosolic class II small heat shock protein gene from *Nelumbo nucifera*, contributes to seed germination vigor and seedling thermotolerance in transgenic Arabidopsis. *Plant cell Rep.* 2012 Feb;31:379-89.
85. Cheng LB, Yang JJ, Yin L, Hui LC, Qian HM, Li SY, Li LJ. Transcription factor NnDREB1 from lotus improved drought tolerance in transgenic *Arabidopsis thalian*a. *Biol. Plant.* 2017 Dec;61(4):651-8.
86. Zhou P, Jiang H, Li J, Jin Q, Wang Y, Xu Y. Genome-wide identification reveals that BZR1 family transcription factors involved in hormones and abiotic stresses response of lotus (Nelumbo). *Horticulturae.* 2023 Aug 3;9(8):882.
87. Cao J, Jin Q, Kuang J, Wang Y, Xu Y. Regulation of flowering timing by ABA-NnSnRK1 signaling pathway in lotus. *Int. J. Mol. Sci.* 2021 Apr 10;22(8):3932.
88. Lin Z, Cao D, Damaris RN, Yang P. Genome-wide identification of MADS-box gene family in sacred lotus (*Nelumbo nucifera*) identifies a SEPALLATA homolog gene involved in floral development. *BMC Plant Biol.* 2020 Dec;20(1):1-5.
89. Cao D, Lin Z, Huang L, Damaris RN, Yang P. Genome-wide analysis of AP2/ERF superfamily in lotus (*Nelumbo nucifera*) and the association between NnADAP and rhizome morphology. *BMC Genom*. 2021 Dec;22:1-2.
90. Kong DZ, Shen XY, Guo B, Dong JX, Li YH, Liu YP. Cloning and expression of an APETALA1-like gene from *Nelumbo nucifera*. *Genet. Mol. Res.* 2015 Jun 18;14(2):6819-29.
91. Hong Y, Yang LW, Li ML, Dai SL. Comparative analyses of light-induced anthocyanin accumulation and gene expression between the ray florets and leaves in chrysanthemum. *Plant Physiol. Biochem.* 2016 Jun 1;103:120-32.
92. Hong Y, Tang X, Huang H, Zhang Y, Dai S. Transcriptomic analyses reveal species-specific light-induced anthocyanin biosynthesis in chrysanthemum. *BMC Genom.* 2015 Dec;16(1):1-8.
93. Xiang L, Liu X, Li H, Yin X, Grierson D, Li F, Chen K. *CmMYB# 7*, an R3 MYB transcription factor, acts as a negative regulator of anthocyanin biosynthesis in chrysanthemum. *J. Exp. Bot.* 2019 Jun 1;70(12):3111-23.
94. Zhao W, Ding L, Liu J, Zhang X, Li S, Zhao K, Guan Y, Song A, Wang H, Chen S, Jiang J. Regulation of lignin biosynthesis by an atypical bHLH protein CmHLB in Chrysanthemum. *J. Exp. Bot.* 2022 Apr 18;73(8):2403-19.
95. Satoh S, Watanabe M, Chisaka K, Narumi T. Suppressed leaf senescence in Chrysanthemum transformed with a mutated ethylene receptor gene mDG-ERS1 (etr1-4). *J. Plant Biol.* 2008 Nov;51:424-7.
96. Narumi T, Kanno Y, Suzuki M, Kishimoto S, Ohmiya A, Satoh S. Cloning of a cDNA encoding an ethylene receptor (DG-ERS1) from chrysanthemum and comparison of its mRNA level in ethylene-sensitive and-insensitive cultivars. *Postharvest Biol. Technol.* 2005 Apr 1;36(1):21-30.
97. Fan Q, Song A, Jiang J, Zhang T, Sun H, Wang Y, Chen S, Chen F. *CmWRKY1* enhances the dehydration tolerance of chrysanthemum through the regulation of ABA-associated genes. *PLoS One*. 2016 Mar 3;11(3):e0150572.
98. Mekapogu M, Kwon OK, Song HY, Jung JA. Towards the improvement of ornamental attributes in chrysanthemum: recent progress in biotechnological advances. *Int. J. Mol. Sci.* 2022 Oct 14;23(20):12284.
99. Nie J, Wen C, Xi L, Lv S, Zhao Q, Kou Y, Ma N, Zhao L, Zhou X. The AP2/ERF transcription factor CmERF053 of chrysanthemum positively regulates shoot branching, lateral root, and drought tolerance. *Plant cell Rep.* 2018 Jul;37:1049-60.
100. Xu Y, Zhao X, Aiwaili P, Mu X, Zhao M, Zhao J, Cheng L, Ma C, Gao J, Hong B. A zinc finger protein BBX19 interacts with ABF3 to affect drought tolerance negatively in chrysanthemum. *Plant Journal.* 2020 Aug;103(5):1783-95.
101. Wang T, Wei Q, Wang Z, Liu W, Zhao X, Ma C, Gao J, Xu Y, Hong B. *CmNF‐YB8* affects drought resistance in chrysanthemum by altering stomatal status and leaf cuticle thickness. *J. Integr. Plant Biol.* 2022 Mar;64(3):741-55.
102. Bai H, Liao X, Li X, Wang B, Luo Y, Yang X, Tian Y, Zhang L, Zhang F, Pan Y, Jiang B. DgbZIP3 interacts with DgbZIP2 to increase the expression of *DgPOD* for cold stress tolerance in chrysanthemum. *Hortic. Res.* 2022;9:uhac105.
103. Takatsu Y, Nishizawa Y, Hibi T, Akutsu K. Transgenic chrysanthemum (*Dendranthema grandiflorum* (Ramat.) Kitamura) expressing a rice chitinase gene shows enhanced resistance to gray mold (*Botrytis cinerea*). *Sci. Hort.* 1999 Dec 1;82(1-2):113-23.
104. Xu G, Chen S, Chen F. Transgenic chrysanthemum plants expressing a harpin Xoo gene demonstrate induced resistance to Alternaria leaf spot and accelerated development. *Russ. J. Plant Physiol.* 2010 Jul;57:548-53.
105. Yu M, Liu Z, Chen S, Chen F. Expression of P. mume PGIP gene in transgenic *Dendranthema morifolium* increased tolerance to disease resistance. *Acta Bot. Sin.* 2010;30(6):1111-6.
106. Yang T, Stoopen G, Thoen M, Wiegers G, Jongsma MA. Chrysanthemum expressing a linalool synthase gene ‘smells good’, but ‘tastes bad’to western flower thrips. Plant Biotechnology Journal. 2013 Sep;11(7):875-82.
107. Xin J, Liu Y, Li H, Chen S, Jiang J, Song A, Fang W, Chen F. CmMLO17 and its partner CmKIC potentially support *Alternaria alternata* growth in Chrysanthemum morifolium. *Hortic. Res.* 2021 Dec 1;8.
108. Miao W, Xiao X, Wang Y, Ge L, Yang Y, Liu Y, Liao Y, Guan Z, Chen S, Fang W, Chen F. CmWRKY6-1–CmWRKY15-like transcriptional cascade negatively regulates the resistance to Fusarium oxysporum infection in Chrysanthemum morifolium. *Hortic. Res.* 2023:uhad101.
109. Bi M, Li X, Yan X, Liu D, Gao G, Zhu P, Mao H. Chrysanthemum WRKY15-1 promotes resistance to *Puccinia horiana* Henn. via the salicylic acid signaling pathway. *Hortic. Res.* 2021;8.
110. Li F, Zhang Y, Tian C, Wang X, Zhou L, Jiang J, Wang L, Chen F, Chen S. Molecular module of CmMYB15‐like‐Cm4CL2 regulating lignin biosynthesis of chrysanthemum (*Chrysanthemum morifolium*) in response to aphid (*Macrosiphoniella sanborni*) feeding. *New Phytol.* 2023 Mar;237(5):1776-93.
111. Shulga OA, Mitiouchkina TY, Shchennikova AV, Skryabin KG, Dolgov SV. Overexpression of AP1-like genes from Asteraceae induces early-flowering in transgenic Chrysanthemum plants. *In Vitro Cell. Dev. Biol. Plant.* 2011 Oct;47:553-60.
112. Sun J, Wang H, Ren L, Chen S, Chen F, Jiang J. CmFTL2 is involved in the photoperiod-and sucrose-mediated control of flowering time in chrysanthemum. *Hortic. Res.* 2017 Dec 27;4.
113. Morita S, Murakoshi Y, Hojo A, Chisaka K, Harada T, Satoh S. Early flowering and increased expression of a FLOWERING LOCUS T-like gene in chrysanthemum transformed with a mutated ethylene receptor gene mDG-ERS1 (etr1-4). *J. Plant Biol.* 2012 Oct;55:398-405.
114. Cheng H, Zhang J, Zhang Y, Si C, Wang J, Gao Z, Cao P, Cheng P, He Y, Chen S, Chen F. The Cm14-3-3μ protein and CCT transcription factor CmNRRa delay flowering in chrysanthemum. *J. Exp. Bot.* 2023 Apr 5:erad130.
115. Liu L, Xue Y, Luo J, Han M, Liu X, Jiang T, Zhao Y, Xu Y, Ma C. Developing a UV–visible reporter‐assisted CRISPR/Cas9 gene editing system to alter flowering time in *Chrysanthemum indicum*. *Plant Biotechnol. J.* 2023 https://doi.org/10.1111/pbi.14062.
116. Lyu J, Aiwaili P, Gu Z, Xu Y, Zhang Y, Wang Z, Huang H, Zeng R, Ma C, Gao J, Zhao X. Chrysanthemum MAF2 regulates flowering by repressing gibberellin biosynthesis in response to low temperature. *Plant Journal.* 2022 Dec;112(5):1159-75.
117. Sumitomo K, Nakano Y, Hisamatsu T, Oda A, Narumi-Kawasaki T, Fukai S, Higuchi Y. Delayed flowering due to ‘cold memory’is regulated by suppression of FLOWERING LOCUS T-like 3 gene in chrysanthemums. *J. Hortic. Sci. Biotechnol.* 2023 May 4;98(3):334-41.
118. Higuchi Y, Narumi T, Oda A, Nakano Y, Sumitomo K, Fukai S, Hisamatsu T. The gated induction system of a systemic floral inhibitor, antiflorigen, determines obligate short-day flowering in chrysanthemums. *Proc. Natl. Acad. Sci. U.S.A.* 2013 Oct 15;110(42):17137-42.
119. Wei Q, Ma C, Xu Y, Wang T, Chen Y, Lü J, Zhang L, Jiang CZ, Hong B, Gao J. Control of chrysanthemum flowering through integration with an aging pathway. *Nat. Commun.* 2017 Oct 10;8(1):829.
120. Wang L, Sun J, Ren L, Zhou M, Han X, Ding L, Zhang F, Guan Z, Fang W, Chen S, Chen F. CmBBX8 accelerates flowering by targeting CmFTL1 directly in summer chrysanthemum. *Plant Biotechnol. J.* 2020;18(7):1562-72.
121. Jiang B, Miao H, Chen S, Zhang S, Chen F, Fang W. The lateral suppressor-like gene, DgLsL, alternated the axillary branching in transgenic chrysanthemum (Chrysanthemum× morifolium) by modulating IAA and GA content. *Plant Mol. Biol. Rep.* 2010 Mar;28:144-51.
122. Yang Q, Cong T, Yao Y, Cheng T, Yuan C, Zhang Q. KNOX Genes Were Involved in Regulating Axillary Bud Formation of Chrysanthemum× morifolium. *Int. J. Mol. Sci.* 2023 Apr 11;24(8):7081.
123. Zhang C, Wei L, Wang W, Qi W, Cao Z, Li H, Bao M, He Y. Identification, characterization and functional analysis of AGAMOUS subfamily genes associated with floral organs and seed development in Marigold (*Tagetes erecta*). *BMC Plant Biol.* 2020 Dec;20:1-7.
124. Yuan C, Huang D, Yang Y, Sun M, Cheng T, Wang J, Pan H, Zhang Q. CmCYC2-like transcription factors may interact with each other or bind to the promoter to regulate floral symmetry development in *Chrysanthemum morifolium*. *Plant Mol. Biol.* 2020 May;103:159-71.
125. Dierck R, Leus L, Dhooghe E, Van Huylenbroeck J, De Riek J, Van Der Straeten D, De Keyser E. Branching gene expression during chrysanthemum axillary bud outgrowth regulated by strigolactone and auxin transport. *Plant Growth Regul*. 2018 Sep;86:23-36.
126. Ishak A, Dong L, Rong H, Zhang S, Zhao L. Isolation and functional analysis of the regulation of branching by isopentenyl transferase gene *CmIPT1* in *Chrysanthemum morifolium* cv.‘Jinba’. *Am. J. Mol. Biol.* 2018 Apr 11;8(2):92-101.
127. Wang J, Guan Y, Ding L, Li P, Zhao W, Jiang J, Chen S, Chen F. The CmTCP20 gene regulates petal elongation growth in *Chrysanthemum morifolium*. *Plant Sci.* 2019 Mar 1;280:248-57.
128. Zhao Y, Broholm SK, Wang F, Rijpkema AS, Lan T, Albert VA, Teeri TH, Elomaa P. TCP and MADS-box transcription factor networks regulate heteromorphic flower type identity in *Gerbera hybrida*. *Plant Physiol.* 2020 Nov 1;184(3):1455-68.
129. Shinoyama H, Sano T, Saito M, Ezura H, Aida R, Nomura Y, Kamada H. Induction of male sterility in transgenic chrysanthemums (Chrysanthemum morifolium Ramat.) by expression of a mutated ethylene receptor gene, Cm-ETR1/H69A, and the stability of this sterility at varying growth temperatures. *Mol. Breed.* 2012 Feb;29:285-95.
130. Kanemaki A, Otani M, Takano M, Fujimoto T, Okuhara H, Nomizu T, Kondo M, Kobayashi H, Tatsuzawa F, Nakano M. Ectopic expression of the R2R3-MYB gene from Tricyrtis sp. results in leaf color alteration in transgenic *Pelargonium crispum.Sci. Hortic.* 2018 Oct 20;240:411-6.
131. Blerot B, Martinelli L, Prunier C, Saint-Marcoux D, Legrand S, Bony A, Sarrabère L, Gros F, Boyer N, Caissard JC, Baudino S. Functional analysis of four terpene synthases in rose-scented Pelargonium cultivars (Pelargonium× hybridum) and evolution of scent in the Pelargonium genus. *Front. Plant Sci.* 2018 Nov 2;9:1435.
132. Mutui TM, Mibus H, Serek M. Influence of thidiazuron, ethylene, abscisic acid and dark storage on the expression levels of ethylene receptors (ETR) and ACC synthase (ACS) genes in Pelargonium. *Plant Growth Regul.* 2007 Nov;53:87-96.
133. Singh P, Pandey SS, Dubey BK, Raj R, Barnawal D, Chandran A, Rahman LU. Salt and drought stress tolerance with increased biomass in transgenic *Pelargonium graveolens* through heterologous expression of ACC deaminase gene from Achromobacter xylosoxidans. *Plant Cell, Tissue Organ Cult.* 2021 Nov;147(2):297-311.
134. Hamama L, Naouar A, Gala R, Voisine L, Pierre S, Jeauffre J, Cesbron D, Leplat F, Foucher F, Dorion N, Hibrand-Saint Oyant L. Overexpression of RoDELLA impacts the height, branching, and flowering behaviour of Pelargonium× domesticum transgenic plants. *Plant Cell Rep.* 2012 Nov;31:2015-29.
135. Gehl C, Wamhoff D, Schaarschmidt F, Serek M. Improved leaf and flower longevity by expressing the etr1-1 allele in *Pelargonium zonale* under control of FBP1 and SAG12 promoters. *Plant Growth Regul.* 2018 Dec;86:351-63.
136. García-Sogo B, Pineda B, Roque E, Antón T, Atarés A, Borja M, Beltrán JP, Moreno V, Cañas LA. Production of engineered long-life and male sterile Pelargonium plants. *BMC Plant Biol.* 2012 Dec;12:1-6.
137. Aida R, Yoshida K, Kondo T, Kishimoto S, Shibata M. Copigmentation gives bluer flowers on transgenic torenia plants with the antisense dihydroflavonol-4-reductase gene. *Plant Sci.* 2000 Dec 7;160(1):49-56.
138. Fukusaki EI, Kawasaki K, Kajiyama SI, An CI, Suzuki K, Tanaka Y, Kobayashi A. Flower color modulations of *Torenia hybrida* by downregulation of chalcone synthase genes with RNA interference. *J. Biotechnol*. 2004 Aug 5;111(3):229-40.
139. Ueyama Y, Suzuki KI, Fukuchi-Mizutani M, Fukui Y, Miyazaki K, Ohkawa H, Kusumi T, Tanaka Y. Molecular and biochemical characterization of torenia flavonoid 3′-hydroxylase and flavone synthase II and modification of flower color by modulating the expression of these genes. *Plant Sci*. 2002 Aug 1;163(2):253-63.
140. Nishihara M, Yamada E, Saito M, Fujita K, Takahashi H, Nakatsuka T. Molecular characterization of mutations in white-flowered torenia plants. *BMC Plant Biol*. 2014 Dec;14:1-3.
141. Junping X, Naing AH, Kim CK. Transcriptional activation of anthocyanin structural genes in Torenia ‘Kauai Rose’via overexpression of anthocyanin regulatory transcription factors. *3 Biotech.* 2018 Nov;8:1-7.
142. Kasajima I, Sasaki K. A chimeric repressor of petunia PH4 R2R3-MYB family transcription factor generates margined flowers in torenia. *Plant Signal. Behav.* 2016 May 3;11(5):e1177693.
143. Barone RP, Knittel DK, Ooka JK, Porter LN, Smith NT, Owens DK. The production of plant natural products beneficial to humanity by metabolic engineering. *Curr. Opin. Plant Biol.* 2020 Dec 1;24:100121.
144. Maeda S, Sasaki K, Kaku H, Kanda Y, Ohtsubo N, Mori M. Overexpression of Rice BSR2 Confers Disease Resistance and Induces Enlarged Flowers in *Torenia fournieri* Lind. *Int. J. Mol. Sci.* 2022 Apr 25;23(9):4735.
145. Sasaki K, Ohtsubo N. Production of multi-petaled *Torenia fournieri* flowers by functional disruption of two class-C MADS-box genes. *Planta.* 2020 May;251:1-6.
146. Sasaki K, Yamaguchi H, Nakayama M, Aida R, Ohtsubo N. Co-modification of class B genes TfDEF and TfGLO in *Torenia fournieri* Lind. alters both flower morphology and inflorescence architecture. *Plant Mol. Biol*. 2014 Oct;86:319-34.
147. Sekiguchi N, Sasaki K, Oshima Y, Mitsuda N. Ectopic expression of AtNF-YA6-VP16 in petals results in a novel petal phenotype in *Torenia fournieri*. *Planta.* 2022 May;255(5):105.
148. Shikata M, Yamaguchi H, Sasaki K, Ohtsubo N. Overexpression of Arabidopsis miR157b induces bushy architecture and delayed phase transition in *Torenia fournieri*. *Planta*. 2012 Oct;236:1027-35.
149. Zhang L, Zhou L, Yung WS, Su W, Huang M. Ectopic expression of *Torenia fournieri* TCP8 and TCP13 alters the leaf and petal phenotypes in *Arabidopsis thaliana*. *Physiol. Plant.* 2021 Nov;173(3):856-66.
150. Yang X, Wang Y, Liu TX, Liu Q, Liu J, Lü TF, Yang RX, Guo FX, Wang YZ. CYCLOIDEA-like genes control floral symmetry, floral orientation, and nectar guide patterning. *Plant Cell*. 2023 May 3:koad115.
151. Su S, Xiao W, Guo W, Yao X, Xiao J, Ye Z, Wang N, Jiao K, Lei M, Peng Q, Hu X. The CYCLOIDEA–RADIALIS module regulates petal shape and pigmentation, leading to bilateral corolla symmetry in *Torenia fournieri* (Linderniaceae). *New Phytol.* 2017 Sep;215(4):1582-93.
152. Sasaki, K., Ohtsubo, N. Production of multi-petaled *Torenia fournieri* flowers by functional disruption of two class-C MADS-box genes. *Planta.* 251, 101 (2020).
153. Naing AH, Kyu SY, Pe PP, Park KI, Lee JM, Lim KB, Kim CK. Silencing of the phytoene desaturase (PDS) gene affects the expression of fruit-ripening genes in tomatoes. *Plant methods*. 2019 Dec;15(1):1-0.
154. Quattrocchio F, Wing J, van der Woude K, Souer E, de Vetten N, Mol J, Koes R. Molecular analysis of the anthocyanin2 gene of petunia and its role in the evolution of flower color. *Plant Cell.* 1999 Aug 1;11(8):1433-44.
155. Napoli C, Lemieux C, Jorgensen R. Introduction of a chimeric chalcone synthase gene into petunia results in reversible co-suppression of homologous genes in trans. *Plant cell.* 1990 Apr 1;2(4):279-89.
156. van Tunen AJ, Mur LA, Brouns GS, Rienstra JD, Koes RE, Mol JN. Pollen-and anther-specific chi promoters from petunia: tandem promoter regulation of the chiA gene. *Plant cell.* 1990 May 1;2(5):393-401.
157. Holton TA, Brugliera F, Tanaka Y. Cloning and expression of flavonol synthase from *Petunia hybrida*. *Plant Journal.* 1993 Dec;4(6):1003-10.
158. Brugliera F, Barri‐Rewell G, Holton TA, Mason JG. Isolation and characterization of a flavonoid 3′‐hydroxylase cDNA clone corresponding to the Ht1 locus of *Petunia hybrida*. *Plant Journal.* 1999 Aug;19(4):441-51.
159. Mori S, Kobayashi H, Hoshi Y, Kondo M, Nakano M. Heterologous expression of the flavonoid 3′, 5′-hydroxylase gene of Vinca major alters flower color in transgenic *Petunia hybrida*. *Plant Cell Rep*. 2004 Jan;22:415-21.
160. Chu YX, Chen HR, Wu AZ, Cai R, Pan JS. Expression analysis of dihydroflavonol 4-reductase genes in *Petunia hybrida*. *Genet. Mol. Res.* 2015 Oct;14(2):5010-21.
161. Holton TA, Cornish EC. Genetics and biochemistry of anthocyanin biosynthesis. *Plant Cell*. 1995 Jul;7(7):1071.
162. Albert NW, Lewis DH, Zhang H, Schwinn KE, Jameson PE, Davies KM. Members of an R2R3‐MYB transcription factor family in Petunia are developmentally and environmentally regulated to control complex floral and vegetative pigmentation patterning. *Plant Journal*. 2011 Mar;65(5):771-84.
163. Colquhoun TA, Kim JY, Wedde AE, Levin LA, Schmitt KC, Schuurink RC, Clark DG. PhMYB4 fine-tunes the floral volatile signature of Petunia× hybrida through PhC4H. *J. Exp. Bot.* 2011 Jan 1;62(3):1133-43.
164. Nadakuduti SS, Uebler JB, Liu X, Jones AD, Barry CS. Characterization of trichome-expressed BAHD acyltransferases in Petunia axillaris reveals distinct acylsugar assembly mechanisms within the Solanaceae. *Plant Physiol*. 2017 Sep 1;175(1):36-50.
165. Spitzer-Rimon B, Farhi M, Albo B, Cna’ani A, Ben Zvi MM, Masci T, Edelbaum O, Yu Y, Shklarman E, Ovadis M, Vainstein A. The R2R3-MYB–like regulatory factor EOBI, acting downstream of EOBII, regulates scent production by activating ODO1 and structural scent-related genes in petunia. *Plant Cell.* 2012 Dec 1;24(12):5089-105.
166. Verdonk JC, Haring MA, Van Tunen AJ, Schuurink RC. ODORANT1 regulates fragrance biosynthesis in petunia flowers. *Plant Cell*. 2005 May 1;17(5):1612-24.
167. Colquhoun TA, Schimmel BC, Kim JY, Reinhardt D, Cline K, Clark DG. A petunia chorismate mutase specialized for the production of floral volatiles. *Plant Journal*. 2010 Jan;61(1):145-55.
168. Liu J, Li J, Wang H, Fu Z, Liu J, Yu Y. Identification and expression analysis of ERF transcription factor genes in petunia during flower senescence and in response to hormone treatments. *J. Exp. Bot.* 2011 Jan 1;62(2):825-40.
169. Wang H, Stier G, Lin J, Liu G, Zhang Z, Chang Y, Reid MS, Jiang CZ. Transcriptome changes associated with delayed flower senescence on transgenic petunia by inducing expression of etr1-1, a mutant ethylene receptor. *PloS One*. 2013 Jul 9;8(7):e65800.
170. Waterland NL. Characterization of the cysteine protease, PhCP10, during the senescence of Petunia x hybrida flowers. *Knowlwdge Bank.* 2007. http://hdl.handle.net/1811/24695.
171. Xu J, Kang BC, Naing AH, Bae SJ, Kim JS, Kim H, Kim CK. CRISPR/Cas9‐mediated editing of 1‐aminocyclopropane‐1‐carboxylate oxidase1 enhances Petunia flower longevity. *Plant Biotechnol. J.* 2020 Jan;18(1):287-97.
172. Sasse J, Schlegel M, Borghi L, Ullrich F, Lee M, Liu GW, Giner JL, Kayser O, Bigler L, Martinoia E, Kretzschmar T. Petunia hybrida PDR2 is involved in herbivore defense by controlling steroidal contents in trichomes. *Plant Cell Environ.* 2016 Dec;39(12):2725-39.
173. Rijpkema AS, Zethof J, Gerats T, Vandenbussche M. The petunia AGL6 gene has a SEPALLATA‐like function in floral patterning. *Plant Journal*. 2009 Oct;60(1):1-9.
174. Matsubara K, Shimamura K, Kodama H, Kokubun H, Watanabe H, Basualdo IL, Ando T. Green corolla segments in a wild Petunia species caused by a mutation in FBP2, a SEPALLATA-like MADS box gene. *Planta*. 2008 Aug;228:401-9.
175. Maes T, Van de Steene N, Zethof J, Karimi M, D’Hauw M, Mares G, Van Montagu M, Gerats T. Petunia Ap2-like genes and their role in flower and seed development. *Plant Cell.* 2001 Feb 1;13(2):229-44.
176. Drummond RS, Martínez-Sánchez NM, Janssen BJ, Templeton KR, Simons JL, Quinn BD, Karunairetnam S, Snowden KC. Petunia hybrida CAROTENOID CLEAVAGE DIOXYGENASE7 is involved in the production of negative and positive branching signals in petunia. *Plant Physiol*. 2009 Dec 1;151(4):1867-77.
177. Tran Tl, Ho Th, Nguyen Dt. Overexpression of the IbOr gene from sweet potato (*Ipomea batatas* Hoang Long') in maize increases total carotenoid and ß-carotene contents. *Turk. J. Biol.* 2017;41(6):1003-10.
178. Tanaka Y, Brugliera F, Chandler S. Recent progress of flower colour modification by biotechnology. *Int. J. Mol. Sci.* 2009 Dec 15;10(12):5350-69.
179. Zufall RA, Rausher MD. The genetic basis of a flower color polymorphism in the common morning glory (*Ipomoea purpurea*). *J. Hered.* 2003 Nov 1;94(6):442-8.
180. Watanabe K, Kobayashi A, Endo M, Sage-Ono K, Toki S, Ono M. CRISPR/Cas9-mediated mutagenesis of the dihydroflavonol-4-reductase-B (DFR-B) locus in the Japanese morning glory Ipomoea (Pharbitis) nil. *Sci. Rep*. 2017 Aug 30;7(1):10028.
181. Boase MR, Lewis DH, Davies KM, Marshall GB, Patel D, Schwinn KE, Deroles SC. Isolation and antisense suppression of flavonoid 3', 5'-hydroxylase modifies flower pigments and colour in cyclamen. BMC Plant Biol. 2010 Dec;10:1-2.
182. Johzuka-Hisatomi Y, Hoshino A, Mori T, Habu Y, Iida S. Characterization of the chalcone synthase genes expressed in flowers of the common and Japanese morning glories. *Genes Genet. Syst.* 1999;74(4):141-7.
183. Kou M, Li C, Song W, Shen Y, Tang W, Zhang Y, Wang X, Yan H, Gao R, Ahmad MQ, Li Q. Identification and functional characterization of a flavonol synthase gene from sweet potato [*Ipomoea batatas* (L.) Lam.]. *Front. Plant Sci.* 2023 May 10;14:1181173.
184. Yamamizo C, Kishimoto S, Ohmiya A. Carotenoid composition and carotenogenic gene expression during Ipomoea petal development. *J. Exp. Bot.* 2010 Mar 1;61(3):709-19.
185. Hoshino A, Mizuno T, Shimizu K, Mori S, Fukada-Tanaka S, Furukawa K, Ishiguro K, Tanaka Y, Iida S. Generation of yellow flowers of the Japanese morning glory by engineering its flavonoid biosynthetic pathway toward aurones. *Plant Cell Physiol.* 2019 Aug 1;60(8):1871-9.
186. Shibuya K, Watanabe K, Ono M. CRISPR/Cas9-mediated mutagenesis of the EPHEMERAL1 locus that regulates petal senescence in Japanese morning glory. *Plant Physiol. Biochem.* 2018 Oct 1;131:53-7.
187. Ding N, Wang A, Zhang X, Wu Y, Wang R, Cui H, Huang R, Luo Y. Identification and analysis of glutathione S-transferase gene family in sweet potato reveal divergent GST-mediated networks in aboveground and underground tissues in response to abiotic stresses. *BMC Plant Biol.* 2017 Dec;17(1):1-5.
188. Prusińska JM, Boniecka J, Dąbrowska GB, Goc A. Identification and characterization of the Ipomoea nil RelA/SpoT Homologs (InRSHs) and potential directions of their transcriptional regulation. *Plant Sci.* 2019 Jul 1;284:161-76.
189. Yamada T, Ichimura K, Kanekatsu M, van Doorn WG. Gene expression in opening and senescing petals of morning glory (*Ipomoea nil*) flowers. *Plant cell Rep*. 2007 Jun;26:823-35.
190. Miyagawa N, Miyahara T, Okamoto M, Hirose Y, Sakaguchi K, Hatano S, Ozeki Y. Dihydroflavonol 4-reductase activity is associated with the intensity of flower colors in delphinium. *Plant Biotechnol. J*. 2015 Sep 25;32(3):249-55.
191. Sakaguchi K, Isobe C, Fujita K, Ozeki Y, Miyahara T. Production of novel red-purple delphinium flowers containing cyanidin-based anthocyanin using hybridization breeding. *J. Hortic.* 2019;88(4):514-20.
192. Ishii I, Sakaguchi K, Fujita K, Ozeki Y, Miyahara T. A double knockout mutant of acyl-glucose-dependent anthocyanin glucosyltransferase genes in Delphinium grandiflorum. *J. Plant Physiol*. 2017 Sep 1;216:74-8.
193. Nishizaki Y, Yasunaga M, Okamoto E, Okamoto M, Hirose Y, Yamaguchi M, Ozeki Y, Sasaki N. p-Hydroxybenzoyl-glucose is a zwitter donor for the biosynthesis of 7-polyacylated anthocyanin in Delphinium. *Plant Cell*. 2013 Oct 1;25(10):4150-65.
194. Matsuba Y, Sasaki N, Tera M, Okamura M, Abe Y, Okamoto E, Nakamura H, Funabashi H, Takatsu M, Saito M, Matsuoka H. A novel glucosylation reaction on anthocyanins catalyzed by acyl-glucose–dependent glucosyltransferase in the petals of carnation and delphinium. *Plant Cell*. 2010 Oct 1;22(10):3374-89.
195. Nishizaki Y, Sasaki N, Yasunaga M, Miyahara T, Okamoto E, Okamoto M, Hirose Y, Ozeki Y. Identification of the glucosyltransferase gene that supplies the p-hydroxybenzoyl-glucose for 7-polyacylation of anthocyanin in delphinium. *J. Exp. Bot.* 2014 Jun 1;65(9):2495-506.
196. Kuroda S, Hakata M, Hirose Y, Shiraishi M, Abe S. Ethylene production and enhanced transcription of an ethylene receptor gene, ERS1, in Delphinium during abscission of florets. *Plant Physiol. Biochem.* 2003 Sep 1;41(9):812-20.
197. Tanase K, Ichimura K. Expression of ethylene receptors Dl-ERS1-3 and Dl-ERS2, and ethylene response during flower senescence in Delphinium. *J. Plant Physiol.* 2006 Nov 1;163(11):1159-66.
198. Abe S, Kuroda S, Hirose Y, Davies E. Molecular basis of ethylene signal transduction and control of abscission of flowers in Delphinium. *Floric. ornam. biotechnol*. 2006:109-23.
199. Kuroda S, Hirose Y, Shiraishi M, Davies E, Abe S. Co-expression of an ethylene receptor gene, ERS1, and ethylene signaling regulator gene, CTR1, in Delphinium during abscission of florets. *Plant Physiol. Biochem*. 2004 Sep 1;42(9):745-51.
200. Okamoto M, Niki T, Azuma M, Shibuya K, Ichimura K. Expression of ethylene biosynthesis genes in the gynoecium and receptacle associated with sepal abscission during senescence in *Delphinium grandiflorum*. *Plant Growth Regul.* 2022 Jul;97(3):593-609.
201. Nitarska D, Boehm R, Debener T, Lucaciu RC, Halbwirth H. First genome edited poinsettias: targeted mutagenesis of flavonoid 3′-hydroxylase using CRISPR/Cas9 results in a colour shift. *Plant Cell Tissue Organ Cult*. 2021 Oct;147(1):49-60.\
202. Clarke JL, Spetz C, Haugslien S, Xing S, Dees MW, Moe R, Blystad DR. Agrobacterium tumefaciens-mediated transformation of poinsettia, *Euphorbia pulcherrima*, with virus-derived hairpin RNA constructs confers resistance to Poinsettia mosaic virus. *Plant cell Rep*. 2008 Jun;27:1027-38.
203. Islam MA, Lütken H, Haugslien S, Blystad DR, Torre S, Rolcik J, Rasmussen SK, Olsen JE, Clarke JL. Overexpression of the AtSHI gene in poinsettia, *Euphorbia pulcherrima*, results in compact plants. *PLoS One*. 2013 Jan 7;8(1):e53377.
204. Li YM, Zhang KM, Jin HH, Zhu L, Li YH. Isolation and expression analysis of four putative structural genes involved in anthocyanin biosynthesis in *Begonia semperflorens*. *J. Hortic. Sci. Biotechnol*. 2015 Jan 1;90(4):444-50.
205. Xu QL, Dong JL, Gao N, Ruan MY, Jia HY, Zhang L, Wang CY. Transgenic lines of *Begonia maculata* generated by ectopic expression of PttKN1. *Biologia.* 2011 Apr 1;66(2):251-7.
206. Feng G, Wang J, Pan Z, Deng C. Integrative Metabolome and Transcriptome Analyses Reveal the Molecular Mechanism of Yellow-Red Bicolor Formation in *Kalanchoe blossfeldiana* Petals. *Horticulturae*. 2023 Jul 24;9(7):844.
207. Sanikhani M, Mibus H, Stummann BM, Serek M. *Kalanchoe blossfeldiana* plants expressing the Arabidopsis etr1-1 allele show reduced ethylene sensitivity. *Plant Cell Rep*. 2008 Apr;27:729-37.
208. Wang L, Zhu C, Jin L, Xiao A, Duan J, Ma L. A novel gene of *Kalanchoe daigremontiana* confers plant drought resistance. *Sci. Rep.* 2018 Feb 7;8(1):2547.
209. Zakharchenko NS, Rukavtsova EB, Shevchuk TV, Furs OV, Pigoleva SV, Lebedeva AA, Chulina IA, Baidakova LK, Bur’yanov YI. The obtainment and characteristics of *Kalanchoe pinnata* L. plants expressing the artificial gene of the cecropin P1 antimicrobial peptide. *Appl. Biochem. Microbiol.* 2016 Jul;52:421-8.
210. García-Sogo B, Pineda B, Castelblanque L, Antón T, Medina M, Roque E, Torresi C, Beltrán JP, Moreno V, Cañas LA. Efficient transformation of *Kalanchoe blossfeldiana* and production of male-sterile plants by engineered anther ablation. *Plant Cell Rep*. 2010 Jan;29:61-77.
211. Zhu C, Wang L, Chen J, Liu C, Zeng H, Wang H. Over-expression of KdSOC1 gene affected plantlet morphogenesis in *Kalanchoe daigremontiana*. *Sci. Rep.* 2017 Jul 17;7(1):5629.
212. Favero BT, Tan Y, Lin Y, Hansen HB, Shadmani N, Xu J, He J, Müller R, Almeida A, Lütken H. Transgenic *Kalanchoë blossfeldiana*, containing individual rol genes and open reading frames under 35s promoter, exhibit compact habit, reduced plant growth, and altered ethylene tolerance in flowers. *Front. Plant Sci.* 2021 May 7;12:672023.
213. Lütken H, Laura M, Borghi C, Ørgaard M, Allavena A, Rasmussen SK. Expression of KxhKN4 and KxhKN5 genes in *Kalanchoe blossfeldiana* ‘Molly’results in novel compact plant phenotypes: towards a cisgenesis alternative to growth retardants. *Plant Cell Rep*. 2011 Dec;30:2267-79.
214. Yang T, Zhang T, Li Y, Kang Y, Wang P, Liu W, Wang Y, Tian L, Dai J, Zhou Y. Genome-Wide Identification and Expression Analysis of the Chalcone Synthase (CHS) Gene Family in *Dendrobium catenatum*. *Agronomy*. 2023 May 28;13(6):1488.
215. Anggraini R, Febriani AL, Mazieda MN, Al-Yamini TH, Listyorini D. Isolation of dihydroflavonol-4-reductase (DFR) gene in Dendrobium helix cv. Pomeo Brown. *KnE Life Sci*. 2017 Jul 11:213-8.
216. Vishwakarma SK, Singh N, Kumaria S. Genome-wide identification and analysis of the PAL genes from the orchids *Apostasia shenzhenica,* *Dendrobium catenatum* and *Phalaenopsis equestris*. *J. Biomol. Struct. Dyn.* 2023 Mar 4;41(4):1295-308.
217. Yu Z, Zhao C, Zhang G, Teixeira da Silva JA, Duan J. Genome-wide identification and expression profile of TPS gene family in *Dendrobium officinale* and the role of DoTPS10 in linalool biosynthesis. *Int. J. Mol. Sci.* 2020 Jul 30;21(15):5419.
218. Ling H, Zeng X, Guo S. Functional insights into the late embryogenesis abundant (LEA) protein family from *Dendrobium officinale* (Orchidaceae) using an *Escherichia coli* system. *Sci. Rep*. 2016 Dec 22;6(1):39693.
219. Fan H, Cui M, Li N, Li X, Liang Y, Liu L, Cai Y, Lin Y. Genome-wide identification and expression analyses of R2R3-MYB transcription factor genes from two Orchid species. *PeerJ.* 2020 Sep 1;8:e9781.
220. Chang C, Chen YC, Hsu YH, Wu JT, Hu CC, Chang WC, Lin NS. Transgenic resistance to *Cymbidium mosaic* virus in Dendrobium expressing the viral capsid protein gene. *Transgenic Res.* 2005 Feb;14:41-6.
221. Song C, Li G, Dai J, Deng H. Genome-wide analysis of PEBP genes in *Dendrobium huoshanense*: Unveiling the antagonistic functions of FT/TFL1 in flowering time. *Front. genet.* 2021 Jul 9;12:687689.
222. Yang SH, Yu H, Goh CJ. Functional characterisation of a cytokinin oxidase gene DSCKX1 in Dendrobium orchid. *Plant Mol. Biol.* 2003 Jan;51:237-48.\
223. Skipper M, Pedersen KB, Johansen LB, Frederiksen S, Irish VF, Johansen BB. Identification and quantification of expression levels of three FRUITFULL-like MADS-box genes from the orchid *Dendrobium thyrsiflorum* (Reichb. f.). *Plant Sci*. 2005 Sep 1;169(3):579-86.
224. Elibox W, Umaharan P. Inheritance of major spathe colors in *Anthurium andraeanum* Hort. is determined by three major genes. *HortScience*. 2008 Jun 1;43(3):787-91.
225. Collette VE, Jameson PE, Schwinn KE, Umaharan P, Davies KM. Temporal and spatial expression of flavonoid biosynthetic genes in flowers of *Anthurium andraeanum*. *Physiol. Plant.* 2004 Nov;122(3):297-304.
226. Li C, Qiu J, Yang G, Huang S, Yin J. Isolation and characterization of a R2R3-MYB transcription factor gene related to anthocyanin biosynthesis in the spathes of *Anthurium andraeanum* (Hort.). *Plant Cell Rep*. 2016 Oct;35:2151-65.
227. Li C, Qiu J, Huang S, Yin J, Yang G. AaMYB3 interacts with AabHLH1 to regulate proanthocyanidin accumulation in *Anthurium andraeanum* (Hort.)—another strategy to modulate pigmentation. *Hortic. Res*. 2019 Dec 1;6.
228. Li C, Qiu J, Yang G, Huang S, Yin J. Ectopic expression of the *Anthurium andraeanum* (Hort.) *R2R3-MYB* genes AaMYB4 and AaMYB5 enhance the flower color in transgenic tobacco. *Plant Cell Tissue Organ Cult.* 2019 Oct;139:105-17.
229. Wei Q, Xia Q, Wang Y, Chen W, Liu C, Zeng R, Xie L, Yi M, Guo H. Profiling of volatile compounds and associated gene expression in two Anthurium cultivars and their F1 hybrid progenies. *Molecules*. 2021 May 13;26(10):2902.
230. Jiang L, Fu Y, Tian X, Ma Y, Chen F, Wang G. The Anthurium APRR2-like gene promotes photosynthetic pigment accumulation in response to salt stress. *Trop. Plant Biol.* 2022 Mar 1:1-0.
231. Liu HC, Tian DQ, Liu JX, Ma GY, Zou QC, Zhu ZJ. Cloning and functional analysis of a novel ascorbate peroxidase (APX) gene from *Anthurium andraeanum*. *J. Zhejiang Univ. Sci. B.* 2013 Dec;14:1110-20.
232. Jiang L, Tian X, Li S, Fu Y, Xu J, Wang G. The AabHLH35 transcription factor identified from *Anthurium andraeanum* is involved in cold and drought tolerance. *Plants.* 2019 Jul 11;8(7):216.
233. Ma G, Zou Q, Shi X, Tian D, Sheng Q. Ectopic expression of the AaFUL1 gene identified in *Anthurium andraeanum* affected floral organ development and seed fertility in tobacco. *Gene.* 2019 May 15;696:197-205.
234. Fan S, Jian D, Chen J, Chen L. Regeneration of Transgenic *Ficus lyrata* via Indirect Somatic Embryogenesis and Isolation of Variants for Development of New Cultivars. *Horticulturae.* 2023 Apr 24;9(5):530.
235. Zhang L, Routsong R, Strand SE. Greatly enhanced removal of volatile organic carcinogens by a genetically modified houseplant, pothos ivy (*Epipremnum aureum*) expressing the mammalian cytochrome P450 2e1 gene. *Environ. Sci. Technol.* 2018 Dec 19;53(1):325-31.
