# Supplementary figures and images for: Designing of future ornamental crops: a biotechnological driven perspective
Source: Hortic Res. 2023 Sep 25;10(11):uhad192. doi: 10.1093/hr/uhad192 (PMC10681008; doi:10.1093/hr/uhad192)

# Designing Future Ornamental Crops

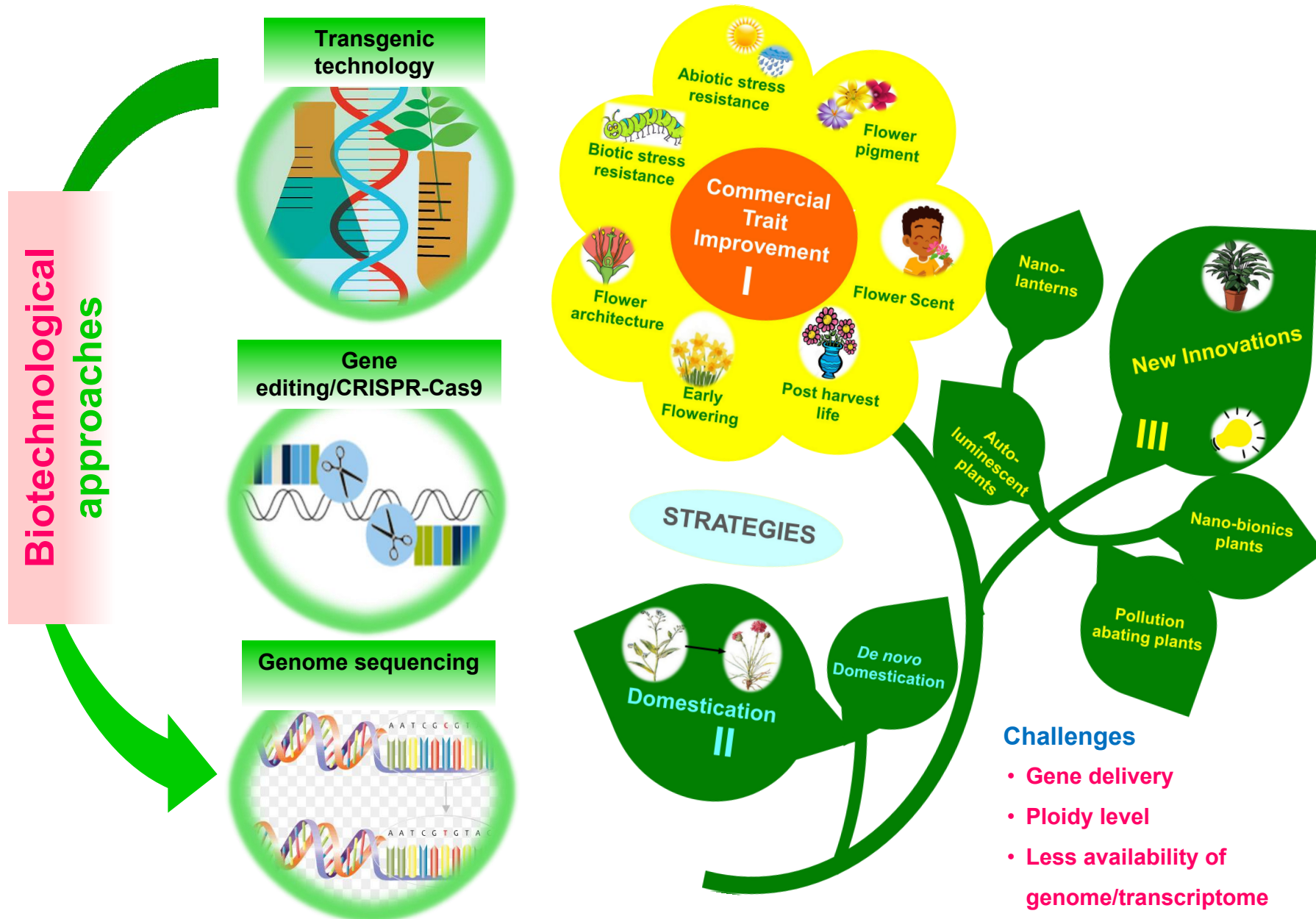

Supplement: Web_Material_uhad192 [file web_material_uhad192.zip › Graphical abstract.pdf]
